# All-Trans Retinoic Acid Attenuates Transmissible Gastroenteritis Virus-Induced Inflammation in IPEC-J2 Cells *via* Suppressing the RLRs/NF‐κB Signaling Pathway

**DOI:** 10.3389/fimmu.2022.734171

**Published:** 2022-01-31

**Authors:** Junning Pu, Daiwen Chen, Gang Tian, Jun He, Zhiqing Huang, Ping Zheng, Xiangbing Mao, Jie Yu, Junqiu Luo, Yuheng Luo, Hui Yan, Bing Yu

**Affiliations:** Key Laboratory for Animal Disease-Resistance Nutrition, Ministry of Education/Institute of Animal Nutrition, Sichuan Agricultural University, Chengdu, China

**Keywords:** all-trans retinoic acid, transmissible gastroenteritis virus, inflammation, IPEC-J2 cells damage, RLRs/NF‐κB pathway

## Abstract

Transmissible gastroenteritis virus (TGEV) infection can cause transmissible gastroenteritis (TGE), especially in suckling piglets, resulting in a significant economic loss for the global pig industry. The pathogenesis of TGEV infection is closely related to intestinal inflammation. All-trans retinoic acid (ATRA) has anti-inflammatory activity and immunomodulatory properties, but it is unclear whether ATRA can attenuate the inflammatory response induced by TGEV. This study aimed to investigate the protective effect of ATRA on TGEV-induced inflammatory injury in intestinal porcine epithelial cells (IPEC-J2) and to explore the underlying molecular mechanism. The results showed that TGEV infection triggered inflammatory response and damaged epithelial barrier integrity in IPEC-J2 cells. However, ATRA attenuated TGEV-induced inflammatory response by inhibiting the release of pro-inflammatory cytokines, including IL-1β, IL-6, IL-8 and TNF-α. ATRA also significantly reversed the reduction of ZO-1 and Occludin protein levels induced by TGEV infection and maintained epithelial barrier integrity. Moreover, ATRA treatment significantly prevented the upregulation of IкBα and NF-κB p65 phosphorylation levels and the nuclear translocation of NF-кB p65 induced by TGEV. On the other hand, treatment of TGEV-infected IPEC-J2 cells with the NF-κB inhibitors (BAY11-7082) significantly decreased the levels of inflammatory cytokines. Furthermore, ATRA treatment significantly downregulated the mRNA abundance and protein levels of TLR3, TLR7, RIG-I and MDA5, and downregulated their downstream signaling molecules *TRIF*, *TRAF6* and *MAVS* mRNA expressions in TGEV-infected IPEC-J2 cells. However, the knockdown of RIG-I and MDA5 but not TLR3 and TLR7 significantly reduced the NF-κB p65 phosphorylation level and inflammatory cytokines levels in TGEV-infected IPEC-J2 cells. Our results indicated that ATRA attenuated TGEV-induced IPEC-J2 cells damage *via* suppressing inflammatory response, the mechanism of which is associated with the inhibition of TGEV-mediated activation of the RLRs/NF‐κB signaling pathway.

## Introduction

Transmissible gastroenteritis (TGE) is a highly contagious enteric disease caused by transmissible gastroenteritis virus (TGEV) infection, with clinical symptoms typified by dehydration, vomiting and severe diarrhea in piglets (1). TGEV is an enveloped, single-stranded, positive-sense RNA virus, belonging to the Coronaviridae family in the order Nidovirales (2). TGEV can infect pigs of various breeds and ages, especially suckling piglets under two weeks of age are the most susceptible, and the mortality rate possibly reaching up to 100%, resulting in significant economic losses to the pig breeding industry (3). Porcine small intestinal epithelial cells are the main target cells of TGEV infection. TGEV replicates in enterocytes covering the villi of the porcine small intestine, causing severe gastroenteritis and impairing the integrity of the gastrointestinal mucosa in infected piglets (4). Consistent with the pathological changes *in vivo*, TGEV infection also induces morphological and biochemical changes of host cells *in vitro*. Previous studies have shown that TGEV could impair the barrier integrity of IPEC-J2 cells by down-regulating the expression of proteins involved in tight and adhesion junctions (5). A large number of studies have shown that the pathogenesis of coronavirus infection is closely related to excessive inflammatory response, which is regulated by a variety of signal cascades, including NF-κB, STAT, MAPKs and so on (6, 7). Previous studies found that the mRNA levels of proinflammatory cytokines IL-1β, IL-6 and TNF-α were significantly increased in the jejunum of piglets infected with TGEV (8). Furthermore, studies have reported that TGEV infection induces inflammation in ST cells and IPEC-J2 cells by activating the NF-κB signaling pathway (9). These evidences indicate that the inflammation of the small intestine may be an important event in the pathogenesis of TGEV infection. At present, vaccination is the main technical means to prevent and treat TGEV infection (10). However, current traditional vaccines, whether inactivated or attenuated, cannot provide full protection for pigs due to the genetic variation of TGEV (11). Therefore, the discovery and development of novel, secure anti-TGEV agents and effective methods for controlling TGEV infection is necessary for the current pig industry.

All-trans retinoic acid (ATRA) is an active metabolite of VA and can regulate a wide range of physiological and biological processes through the action of retinoid acid receptor (RAR) (12). The primary biological functions of ATRA include regulating embryonic development, reproduction, vision, cell growth and differentiation (13, 14). In addition, recent studies have shown that as an immunomodulator, ATRA also plays important roles in regulating immune response and maintaining intestinal homeostasis, especially under stress conditions (15). ATRA has been indicated to be impactful in many intestinal inflammation models (16, 17). He et al. reported that ATRA improves the intestinal barrier function and reverses LPS-induced intestinal barrier damage *via* enhancing the expressions of tight junction proteins (18). Hong et al. reported that ATRA attenuates dextran sulfate sodium (DSS)-induced murine colitis by suppressing NF-κB signaling pathway (19). Penny et al. reported that in the experimental murine IBD model, the blockade of the retinoic acid degrading enzyme CYP26A1 can restore the level of ATRA and reduce intestinal inflammation (20). These results indicated that ATRA has the effect of relieving intestinal inflammation. In addition, previous studies reported that ATRA can attenuate virus-induced inflammatory response (21–23). However, it is unclear whether ATRA can attenuate TGEV-induced intestinal inflammatory damage.

Therefore, this study aimed to investigate the protective effect of ATRA on TGEV-induced inflammatory injury in intestinal porcine epithelial cells (IPEC-J2) and to explore the underlying molecular mechanism. We assumed that ATRA might inhibit inflammatory response by regulating NF-κB signaling pathways, and eventually attenuating TGEV-induced intestinal barrier damage.

## Materials and Methods

### Materials

All-trans retinoic acid (ATRA, ≥98% HPLC) and dimethyl sulfoxide (DMSO) were acquired from Sigma-Aldrich (Shanghai, China). Rabbit anti-ZO-1(61–7300) was purchased from Invitrogen (CA, USA). Rabbit anti-Occludin (ab31721) was purchased from Abcam (Cambridge, UK). Mouse anti-IκBα (4814), mouse anti-NF-κB p65 (6956) and rabbit anti-p-NF-κB p65 (3033) were obtained from Cell signaling Technology (Beverly, USA). Mouse anti-p-IκBα (sc-8404), mouse anti-β-actin antibody (sc-47778) and goat anti-rabbit/mouse IgG -HRP secondary antibody (sc-2030 and sc-2031) were purchased from Santa Cruz Biotechnology (Santa Cruz, USA). The NF-κB inhibitor (BAY11-7082) was purchased from Selleck (Selleckchem, Huston USA).

### Cells Culture and Virus Infection

IPEC-J2 cells and ST cells were obtained from the American Type Culture Collection (ATCC, USA) and cultured in Dulbecco’s Modified Eagle’s Medium and Ham’s F-12 Nutrient Mixture (DMEM/F12, Gibco, Shanghai, China) or Dulbecco’s Modified Eagle’s Medium (DMEM, Gibco, Shanghai, China) supplemented with 10% fetal bovine serum (Gibco, Shanghai, China) and 1% streptomycin and penicillin (Gibco, Shanghai, China), and maintained at 37°C with 5% CO_2_ atmosphere incubator. The medium was changed every 2 days to avoid nutrient depletion. TGEV (SC-T strain) was provided by Prof. Zhiwen Xu, College of Veterinary Medicine, Sichuan Agricultural University. As described previously (24), the TGEV used for IPEC-J2 cells infection was harvested from ST cells and virus titers were determined by 50% tissue culture infective doses (TCID50). For TGEV infection, 70–80% confluent IPEC-J2 cells were inoculated with TGEV [multiplicity of infection (MOI) = 1] at 37°C for 1 h. After TGEV infection, the cells were washed 3 times with PBS and added fresh growth medium. Subsequently, cells and supernatants were harvested at the indicated time points.

### siRNA Transfection of IPEC-J2 Cells

The siRNA targeting porcine TLR-3, TLR-7, RIG-I, MDA5 and negative control siRNA (siNC) were synthesized by Sangon Biotech Co., Ltd (Shanghai, China), and the target sequences were listed in **Table 1**. Cells were transfected with siRNA at indicated concentrations using lipofectamine 3000 (Invitrogen) according to the manufacturer’s instructions. After transfection, the cells were used for subsequent analysis or treatment.

**Table 1:** siRNA target sequences.

Gene name	The target sequence (5’ –3’)
siNC	Sense: UUCUCCGAACGUGUCACGUTT Antisense: ACGUGACACGUUCGGAGAATT
siTLR3	Sense: CAACACAAGCAUUGAGAAUTT Antisense: AUUCUCAAUGCUUGUGUUGTT
siTLR7	Sense: CCUUGGACCUAAGUAGAAATT Antisense: UUUCUACUUAGGUCCAAGGTT
siRIG-I	Sense: GUACAAAGUUGCAGGCAUUTT Antisense: AAUGCCUGCAACUUUGUACTT
siMDA5	Sense: GCAUCUCAGGCCUUACCAATT Antisense: UUGGUAAGGCCUGAGAUGCTT

### Cell Viability Assay

Cell viability was determined using a commercial CCK-8 kit (Beyotime Biotechnology, Shanghai, China) according to the manufacturer’s instructions. Briefly, IPEC-J2 cells were cultured in 96-well plates at a density of 1×10^4^ cells/well and treated with various concentrations of ATRA (0, 1, 10, 20, 40, 60, 80, 100 and 200 μM) at 37°C for 48 h. Then, 10 ul CCK-8 assay solution was added into each well for 2 h at 37°C. Finally, the optical density (OD) of each well at 450 nm was measured using a microplate reader (SpectraMax M2, Molecular Devices, Sunnyvale, CA, USA).

### Lactate Dehydrogenase Activity Assay

To identify cell membrane damage, the activity of lactate dehydrogenase (LDH) in IPEC-J2 cells culture medium was detected by LDH assay kit (Nanjing Jiancheng Bioengineering Institute, Nanjing, China) following the manufacturer’s instructions.

### Cytokine Concentration Assay

The concentrations of IL-1β, IL-6, IL-8 and TNF-α in IPEC-J2 cells culture medium were determined by using the commercially ELISA kits (Jiangsu Meimian Biotechnology Co., Ltd., Jiangsu, China) according to the manufacturer’s instructions.

### RNA Extraction and Real-Time PCR

Total RNA was extracted from IPEC-J2 cells using TRIzol reagent (TaKaRa Biotechnology Co, Ltd, Dalian, China) following the manufacturer’s instructions. The integrity of RNA was checked by electrophoresis on a 1.5% agarose gel. Complementary DNA (cDNA) was synthesized from 1µg of total RNA using the PrimeScripte RT reagent kit (Takara) according to the manufacturer’s instructions. Then, the synthesized cDNA was diluted (1:4) and real-time quantitative PCR amplification was performed using SYBR Premix Ex TaqTM kits (TaKaRa) on the CFX96 Real-Time PCR Detection System (Bio-Rad). The primers were synthesized by TaKaRa Biotechnology Co, Ltd. (Dalian, China), which were listed in **Table 2**. The thermal cycling parameters were as follows: pre-denaturation at 95°C for 30s, then 40 cycles at 95°C for 5 s, 60°C for 34 s and extension at 72°C for 60 s. Each reaction was completed with a melting curve analysis to ensure the specificity of the reaction. The relative mRNA levels of target genes were analyzed using the 2^−ΔΔCt^ method and using *β*-actin as the reference gene (25). All reactions were performed in triplicate.

**Table 2:** Primer sequences used for real-time PCR.

Gene	Primer sequence (5’ –3’)	Product length (bp)	GeneBank accession No.
*TNF-α*	F: CGTGAAGCTGAAAGACAACCAG	121	NM_214022.1
	R: GATGGTGTGAGTGAGGAAAACG		
*IL-1β*	F: CAGCTGCAAATCTCTCACCA	112	NM_214055.1
	R: TCTTCATCGGCTTCTCCACT		
*IL-6*	F: TTCACCTCTCCGGACAAAAC	122	NM_001252429.1
	R: TCTGCCAGTACCTCCTTGCT		
*IL-8*	F: AGTTTTCCTGCTTTCTGCAGCT R: TGGCATCGAAGTTCTGCACT	72	NM_213867.1
*IL-10*	F: CCTGGAAGACGTAATGCCGA R: CACGGCCTTGCTCTTGTTTT	148	NM_214041.1
*RIG-I*	F: AGAGCAGCGGCGGAATC	82	NM_213804.2
	R: GGCCATGTAGCTCAGGATGAA		
*MDA5*	F: TCCGGGAAACAGGCAACTC	75	NM_001100194.1
	R: CAAAGGATGGAGAGGGCAAGT		
*MAVS*	F: TGGGTACAGTCCTTCATCGG R: GGGTAACTTGGCTCATCCTCT	116	NM_020746.5
*TLR2*	F: TCACTTGTCTAACTTATCATCCTCTTG	162	XM_005653576.3
	R: TCAGCGAAGGTGTCATTATTGC		
*TLR3*	F: TGGAAAAAGGAATGGCCAGC R: ACAAGGCAAACTCCTGCTCA	265	NM_001042467.3
*TLR4*	F: TTACAGAAGCTGGTTGCCGT R: TCCAGGTTGGGCAGGTTAGA	152	NM-001293316.1
*TLR7*	F: CAATGGTCCCTGAGCGTTTG R: AGCCTGGTTGAAGACAGCAG	126	NM_016562
*TLR9*	F: CACGACAGCCGAATAGCAC	121	NM_213958.1
	R: GGGAACAGGGAGCAGAGC		
*MyD88*	F: GTGCCGTCGGATGGTAGTG	65	NM001099923
	R: TCTGGAAGTCACATTCCTTGCTT		
*TRIF*	F: CAAGTGGAGGAAGGAACAGG R: CAACTGCGTCTGGTAGGACA	139	XM_003362039.1
*TRAF6*	F: CAAGAGAATACCCAGTCGCACA R: ATCCGAGACAAAGGGGAAGAA	122	NM-001105286.1
*β-actin*	F: GGATGACGATATTGCTGCGC	190	XM_003124280.5
	R: GATGCCTCTCTTGCTCTGGG		

TNF-α, tumor necrosis factor-α; IL, interleukin; RIG-I, retinoic acid-inducible gene I; MDA5, melanoma differentiation associated gene 5; MAVS, mitochondrial antiviral signaling protein; TLR, toll-like receptor; MYD88, myeloid differentiation factor 88; TRIF, TIR domain-containing adaptor inducing interferon β; TRAF6, TNF receptor-associated factor 6

### Western Blot Analysis

Proteins from IPEC-J2 cell samples were acquired using RIPA lysis buffer containing PMSF and phosphatase inhibitor. After incubating at 4°C for 30 min, samples were homogenized and centrifuged at 12,000 g for 15 min at 4°C, and the supernatants were collected. The protein concentrations in the supernatants were determined by a BCA protein assay kit (Thermo Scientific, MA, USA). Then, equal amounts of protein (25 μg) were separated by 10% sodium dodecyl sulphate–polyacrylamide gel electrophoresis (SDS-PAGE) and transferred to polyvinylidene difluoride (PVDF) membranes (Merck Millipore Ltd., Tullagreen, Ireland). The membranes were blocked using 5% nonfat dry milk in 1× TBST for 1 h at room temperature followed by an overnight incubation at 4°C with indicated primary antibodies. Following three flushes with 1× TBST, the membranes were incubated with the corresponding HRP- conjugated secondary antibodies for 1 h at room temperature. Finally, the protein bands were visualized with ECL chemiluminescence kit (Beyotime Biotechnology, Shanghai, China) by the ChemiDoc™ XRS Imager System (Bio-Rad). The protein band densities were quantified using Image Lab software (Bio-Rad).

### Immunofluorescence Assay

IPEC-J2 cells were cultured in a 12-well cell culture plate and infected with TGEV at MOI of 1 for 1 h. Afterwards, the cells were washed with PBS and cultured with 80 μM ATRA for 36 h. After cell treatment, IPEC-J2 cells were fixed with cold 4% paraformaldehyde for 15 min and permeated with 0.05% Triton X-100 for 15 min at room temperature. Then, the IPEC-J2 cells were incubated with primary anti-NF-κB p65 mouse antibodies overnight at 4°C (1:100 dilution, Cell signaling Technology, USA). The cells were then washed with PBS and incubated with Alexa Fluor 488-conjugated goat anti-mouse IgG antibody (Abcam, Shanghai, China) for 2 h at room temperature in the dark. After washing with PBS, the cells were mounted using Vectashield Antifade Mounting Medium with DAPI (Beyotime Biotechnology, Shanghai, China). Images were captured using a fluorescence microscope (Leica DMI 4000 B).

### Statistical Analysis

All data were presented as the means ± standard error of mean (SEM). Statistical differences were analyzed by the unpaired two-tailed Student’s t-test and/or one-way analysis of variance (ANOVA) using SPSS 22.0 statistics software (Chicago, IL, USA). *P* < 0.05 was considered statistically significant.

## Results

### Effects of ATRA on the Viability of IPEC-J2 Cells

**Figure 1:**
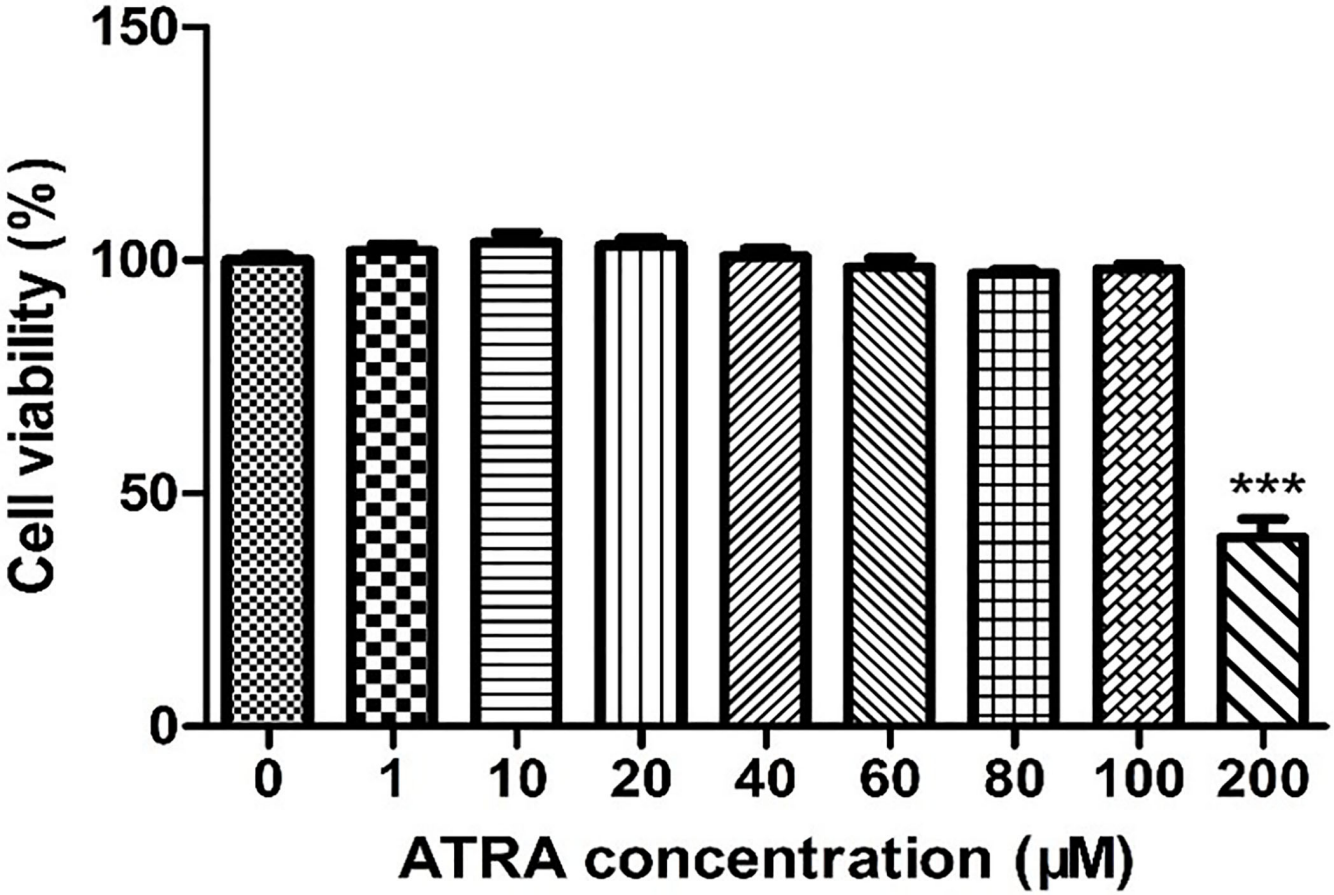
Effects of ATRA on the viability of IPEC-J2 cells. IPEC-J2 cells were treated with various concentrations of ATRA (0, 1, 10, 20, 40, 60, 80, 100 and 200 μM) for 48 h. Cell viability was measured by the CCK-8 assay. Data are presented as means ± SEM (n = 8). ***P < 0.001 compared with the control group.

To investigate the cytotoxic effects of ATRA on IPEC-J2 cells, IPEC-J2 cells were treated with various concentrations of ATRA (0, 1, 10, 20, 40, 60, 80, 100 and 200 μM) for 48 h. As shown in **Figure 1**, ATRA concentrations ≤100 μM did not significantly affect cell viability (*P* > 0.05), but a marked decrease in cell viability was observed at ATRA concentration with 200 μM compared with the control group (*P* < 0.001). Therefore, an ATRA concentration range of 0~80 μM was used in the subsequent experiments.

### ATRA Attenuated TGEV-Induced Inflammatory Response in IPEC-J2 Cells

**Figure 2:**
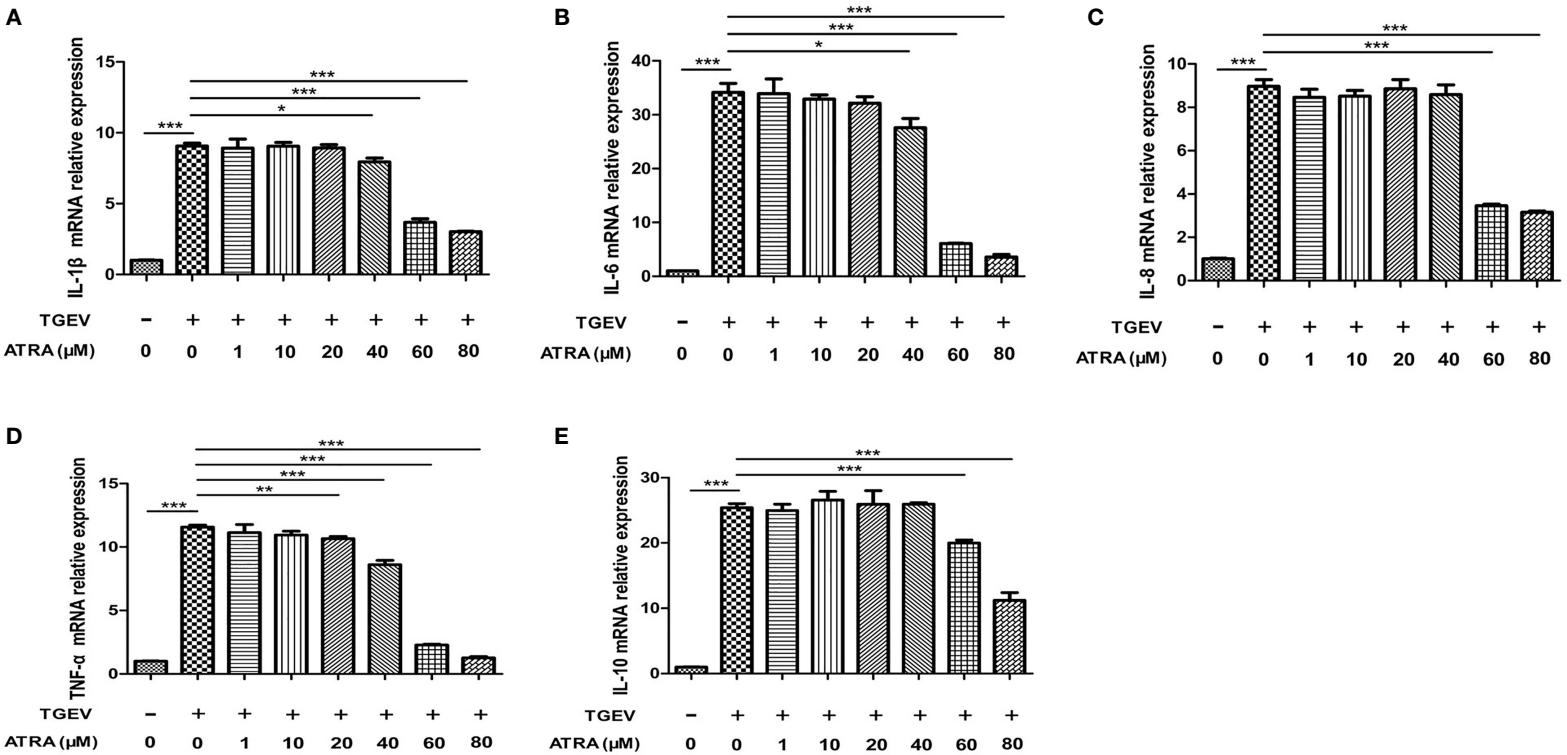
Effects of ATRA on the gene expressions of inflammatory cytokines in TGEV-infected IPEC-J2 cells. The cells were mock-infected or infected with TGEV (1 MOI) for 1 h, followed by incubation with various concentrations of ATRA (0, 1, 10, 20, 40, 60 and 80 μM) for 36 h Total RNA was extracted from cells and the mRNA abundance of *IL-1β* **(A)**, *IL‐6* **(B)**, *IL‐8* **(C)**, *TNF‐α* **(D)** and *IL-10* **(E)** were analyzed by real‐time PCR. Data are presented as means ± SEM (n = 4). *P < 0.05, **P < 0.01 and ***P < 0.001.

**Figure 3:**
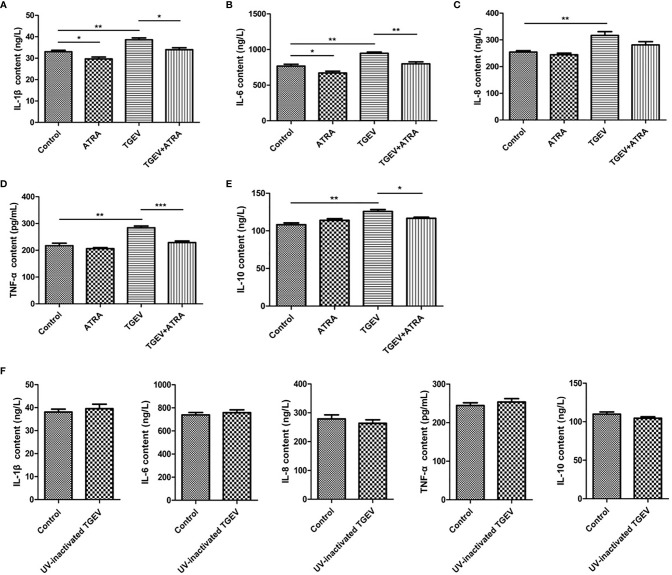
Effects of ATRA on the concentrations of inflammatory cytokines in TGEV-infected IPEC-J2 cells. **(A**–**E)** The cells were mock-infected or infected with TGEV (1 MOI) for 1 h, followed by incubation with or without 80 μM ATRA for 36 h. **(F)** The cells were mock-infected or infected with UV-inactivated TGEV (1 MOI) for 1 h, followed by incubation without ATRA for 36 h. The concentrations of IL‐1β, IL‐6, IL‐8, TNF‐α and IL-10 in IPEC-J2 cells culture medium were measured by ELISA. Data are presented as means ± SEM (n = 4). *P < 0.05, **P < 0.01 and ***P < 0.001.

To assess whether ATRA attenuated the inflammatory response induced by TGEV, the mRNA levels of the inflammatory cytokines were analyzed by real‐time PCR. The results showed that TGEV infection significantly up‐regulated the mRNA levels of *IL-1β*, *IL-6*, *IL-8*, *TNF-α* and *IL-10* (*P* < 0.001), whereas ATRA treatment significantly inhibited the up‐regulation of the gene expressions of inflammatory cytokines in a dose-dependent manner (*P* < 0.05). The highest concentration ATRA (80 μM) was the most effective in decreasing the mRNA expressions of inflammatory cytokines induced by TGEV (*P* < 0.001) (**Figure 2**). To further determine the effects of ATRA on the TGEV‐induced inflammatory response, we detected the concentrations of inflammatory cytokines in IPEC-J2 cells culture medium. Compared with the control group, ATRA treatment significantly decreased the concentrations of IL-1β and IL-6 in IPEC-J2 cells culture medium (*P* < 0.05). Compared with the control group, TGEV infection significantly increased the concentrations of IL-1β, IL-6, IL-8, TNF-α and IL-10 in IPEC-J2 cells culture medium (*P* < 0.01). However, the increase in the concentrations of inflammatory cytokines(IL-1β, IL-6, IL-8, TNF-α and IL-10)was not detected in IPEC-J2 cells culture medium treated with UV-inactivated TGEV (*P* > 0.05). Furthermore, ATRA treatment significantly inhibited the elevation of IL-1β, IL-6, TNF-α and IL-10 concentrations induced by TGEV (*P* < 0.05) (**Figure 3**). These results indicated that TGEV infection could induce the inflammatory response in IPEC-J2 Cells, and this ability required TGEV replication; ATRA treatment could attenuate the inflammatory response induced by TGEV.

### ATRA Attenuated TGEV-Induced Epithelial Barrier Integrity Damage in IPEC-J2 Cells

**Figure 4:**
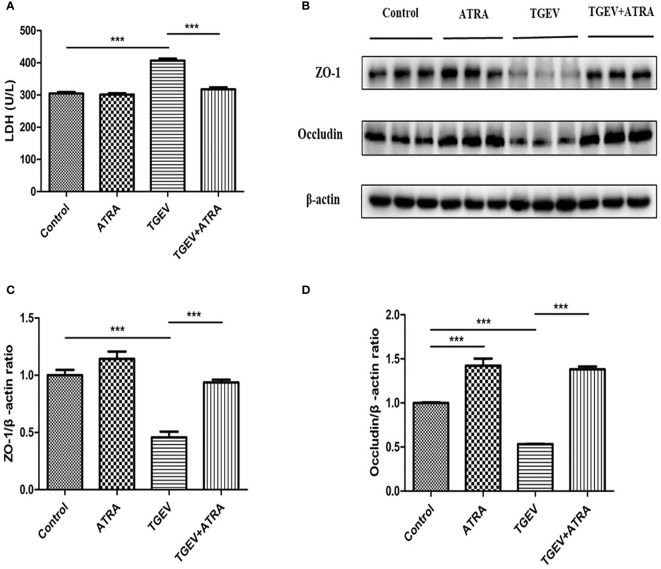
Effects of ATRA on the epithelial barrier integrity in TGEV-infected IPEC-J2 cells. The cells were mock-infected or infected with TGEV (1 MOI) for 1 h, followed by incubation with or without 80 μM ATRA for 36 h. **(A)** LDH Activity. Data are presented as means ± SEM (n = 4). ***P < 0.001. **(B–D)** Western blot and densitometric analysis of ZO-1 and Occludin. Data are presented as means ± SEM (n = 3). ***P < 0.001.

LDH is an important marker for assessing cell permeability. To identify whether ATRA attenuated cell epithelial barrier integrity damage induced by TGEV, LDH activity in IPEC-J2 cell culture medium was detected. As shown in **Figure 4A**, compared with the control group, TGEV infection significantly increased the LDH activity in IPEC-J2 cells culture medium, whereas ATRA treatment significantly decreased LDH released from the TGEV-infected IPEC-J2 cells (*P* < 0.001). In addition, compared with the control group, ATRA treatment significantly increased Occludin protein level (*P* < 0.001). TGEV infection significantly reduced the protein levels of ZO-1 and Occludin compared with the control group (*P* < 0.001). However, ATRA treatment significantly reversed this reduction of ZO-1 and Occludin protein levels induced by TGEV (*P*wfi 2 < 0.001) (**Figures 4B–D**).

### ATRA Prevented TGEV-Induced NF-κB Signaling Pathway Activation in IPEC-J2 Cells

**Figure 5:**
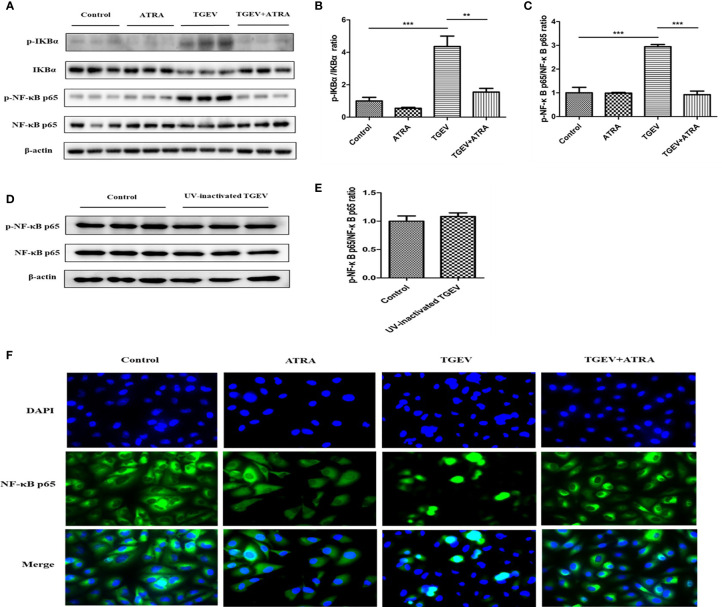
Effects of ATRA on NF-κB signaling pathway in TGEV-infected IPEC-J2 cells. **(A–C, F)** The cells were mock-infected or infected with TGEV (1 MOI) for 1 h, followed by incubation with or without 80 μM ATRA for 36 h. **(D, E)** The cells were mock-infected or infected with UV-inactivated TGEV (1 MOI) for 1 h, followed by incubation without ATRA for 36 h. **(A–E)** The phosphorylation levels of IκBα and NF-κB were analyzed by western blot. Data are presented as means ± SEM (n = 3). **P < 0.01 and ***P < 0.001. **(F)** Immunofluorescence analysis nuclear translocation of NF-κB p65.

Compared with the control group, TGEV infection significantly upregulated the phosphorylation levels of IκBα and NF-κB p65 in IPEC-J2 cells (*P* < 0.001), whereas UV-inactivated TGEV treatment did not induce the upregulation of NF-κB p65 phosphorylation level in IPEC-J2 cells (*P* > 0.05). Furthermore, ATRA treatment significantly prevented the upregulation of IκBα and NF-κB p65 phosphorylation levels induced by TGEV (*P* < 0.01) (**Figures 5A–E**). Further immunofluorescence analysis found that TGEV infection induced the translocation of NF-κB p65 from cytoplasm to nucleus in IPEC-J2 cells, and ATRA treatment inhibited this process (**Figure 5F**). These results indicated that TGEV infection could activate the NF-κB signaling pathway, and this ability required TGEV replication; ATRA treatment could prevent the NF-κB signaling pathway activation induced by TGEV.

### ATRA Attenuated TGEV-Induced Inflammatory Response in IPEC-J2 Cells *via* Suppressing the NF‐κB Signaling Pathway

**Figure 6:**
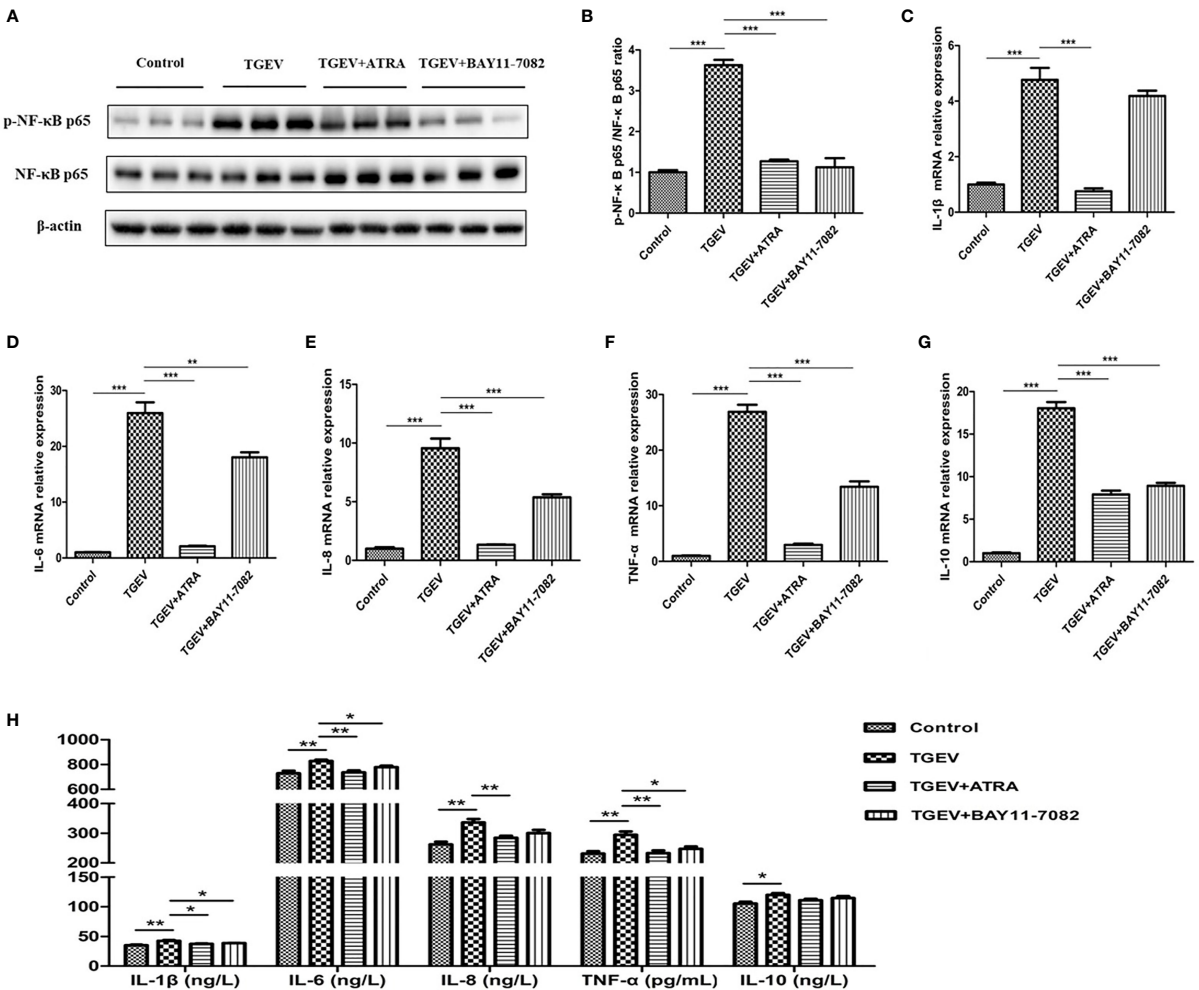
Effects of ATRA and BAY11-7082 on inflammatory response in TGEV-infected IPEC-J2 cells. The cells were pretreated or untreated (exposed to the equal amount of DMSO) with a NF-κB inhibitor (BAY11-7082, 10 μM) for 2 h followed by mock-infection or infection with TGEV (1 MOI) for 1 h, and then incubation with BAY11-7082 or 80 μM ATRA for 36 h. **(A, B)** The phosphorylation level of NF-κB was analyzed by western blot. Data are presented as means ± SEM (n = 3). ***P < 0.001. **(C–G)** the mRNA abundance of *IL‐1β*, *IL‐6*, *IL‐8*, *TNF‐α* and *IL-10* were analyzed by real‐time PCR. Data are presented as means ± SEM (n = 4). ***P < 0.001. **(H)** The concentrations of IL‐1β, IL‐6, IL‐8, TNF‐α and IL-10 in IPEC-J2 cells culture medium were measured by ELISA. Data are presented as means ± SEM (n = 4). *P < 0.05 and **P < 0.01.

To further investigate whether ATRA attenuated TGEV-induced inflammatory response *via* suppressing the NF‐κB signaling pathway, the effect of ATRA was examined by using by NF-κB inhibitors (BAY11-7082). As shown in **Figure 6**, we found that TGEV infection significantly increased the phosphorylation level of NF-κB p65 in IPEC-J2 cells compared with the control group, whereas BAY11-7082 treatment significantly inhibited the enhancing of NF-κB p65 phosphorylation level induced by TGEV (*P* < 0.001). Furthermore, we found that TGEV infection significantly upregulated the mRNA levels of *IL-1β*, *IL-6*, *IL-8*, *TNF-α* and *IL-10* in IPEC-J2 cells and increased the concentrations of IL-1β, IL-6, IL-8, TNF-α and IL-10 in IPEC-J2 cells culture medium compared with the control group (*P* < 0.05). However, BAY11-7082 treatment significantly inhibited the upregulation of *IL-6*, *IL-8*, *TNF-α* and *IL-10* mRNA levels in IPEC-J2 cells and the enhancing of IL-1β, IL-6 and TNF-α concentrations in IPEC-J2 cells culture medium induced by TGEV (*P* < 0.05).

### Effects of ATRA and TGEV on TLRs/RLRs Signaling Pathway in IPEC-J2 Cells

**Figure 7:**
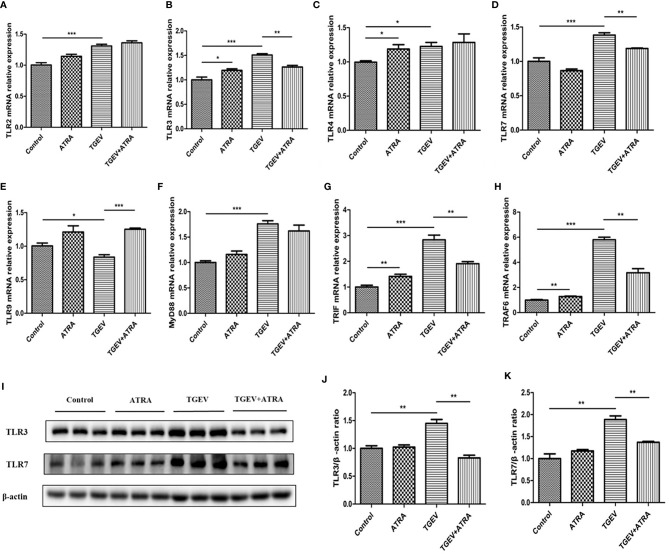
Effects of ATRA and TGEV on TLRs signaling pathway in IPEC-J2 cells. The cells were mock-infected or infected with TGEV (1 MOI) for 1 h, followed by incubation with or without 80 μM ATRA for 36 h. **(A–H)** Total RNA was extracted from cells and the mRNA abundance of *TLR2*, *TLR3*, *TLR4*, *TLR7*, *TLR9*, *MyD88*, *TRIF* and *TRAF6* were analyzed by real‐time PCR. Data are presented as means ± SEM (n = 4). *P < 0.05, **P < 0.01 and ***P < 0.001. **(I–K)** Western blot and densitometric analysis of TLR3 and TLR7. Data are presented as means ± SEM (n = 3). **P < 0.01.

**Figure 8:**
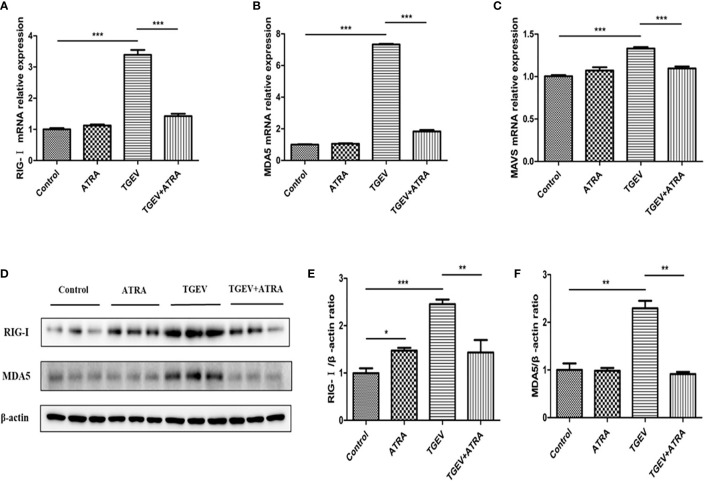
Effects of ATRA and TGEV on RLRs signaling pathway in IPEC-J2 cells. The cells were mock-infected or infected with TGEV (1 MOI) for 1 h, followed by incubation with or without 80 μM ATRA for 36 h. **(A–C)** Total RNA was extracted from cells and the mRNA abundance of *RIG-Ⅰ*, *MDA5* and *MAVS* were analyzed by real‐time PCR. Data are presented as means ± SEM (n = 4). ***P < 0.001. **(D–F)** Western blot and densitometric analysis of RIG-I and MDA5. Data are presented as means ± SEM (n = 3). *P < 0.05, **P < 0.01 and ***P < 0.001.

As shown in **Figure 7**, compared with the control group, ATRA treatment significantly increased the mRNA levels of *TLR3*, *TLR4*, *TRIF* and *TRAF6* (*P* < 0.05). TGEV infection significantly upregulated the mRNA levels of *TLR2*, *TLR3*, *TLR4*, *TLR7*, *MyD88*, *TRIF* and *TRAF6*, and downregulated *TLR9* mRNA level compared with the control group (*P* < 0.05). However, ATRA treatment significantly suppressed the upregulation of *TLR3*, *TLR7*, *TRIF* and *TRAF6* mRNA levels and the downregulation of *TLR9* mRNA level induced by TGEV (*P* < 0.01). Furthermore, TGEV infection significantly increased the protein levels of TLR3 and TLR7 compared with the control group, whereas ATRA treatment significantly inhibited the enhancing of TLR3 and TLR7 protein levels induced by TGEV (*P* < 0.01). As shown in **Figure 8**, the mRNA abundance of *RIG-Ⅰ*, *MDA5* and *MAVS* was significantly upregulated in TGEV group compared with the control group, and ATRA treatment significantly inhibited the upregulation of *RIG-Ⅰ*, *MDA*5 and *MAVS* mRNA abundance induced by TGEV (*P* < 0.001). Furthermore, TGEV infection significantly increased the protein levels of RIG-I and MDA5 compared with the control group, whereas ATRA treatment significantly inhibited the enhancing of RIG-I and MDA5 protein levels induced by TGEV (*P* < 0.01).

### ATRA Attenuated TGEV-Induced Inflammatory Response in IPEC-J2 Cells *via* Suppressing the RLRs/NF‐κB Signaling Pathway

**Figure 9:**
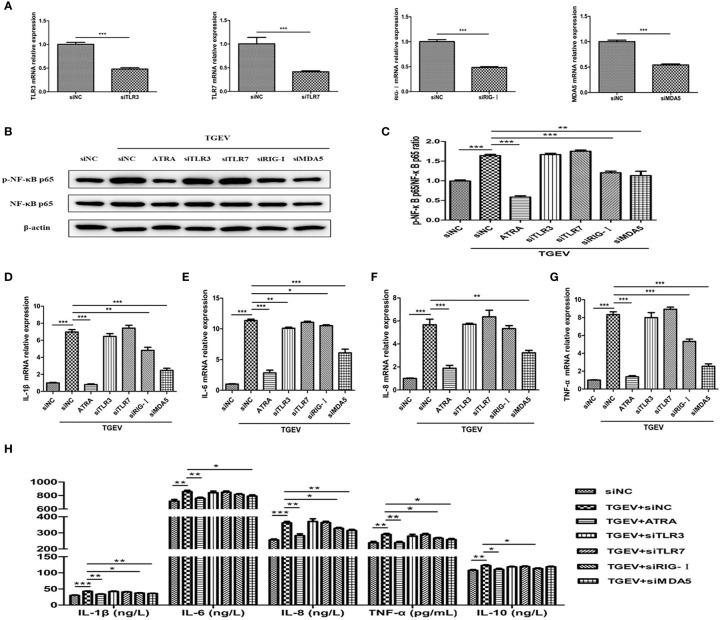
ATRA attenuated TGEV-induced inflammatory response in IPEC-J2 cells *via* suppressing the RLRs/NF‐κB signaling pathway. **(A)** The cells were transfected with 100 nM specific siRNAs targeting TLR3, TLR7, RIG-I or MDA5, or negative control siRNA (siNC) for 24 h, then cells were collected and the mRNA abundance of *TLR3*, *TLR7*, *RIG-I* and *MDA5* were analyzed by real‐time PCR. **(B–G)** The cells were transfected with 100 nM specific siRNAs targeting TLR3, TLR7, RIG-I or MDA5, or negative control siRNA (siNC) for 24 h followed by mock-infection or infection with TGEV (1 MOI) for 1 h, and then incubation with or without 80 μM ATRA for 36 h. **(B, C)** The phosphorylation level of NF-κB was analyzed by western blot. Data are presented as means ± SEM (n = 3). **P < 0.01 and ***P < 0.001. **(D–G)** the mRNA abundance of *IL‐1β*, *IL‐6*, *IL‐8* and *TNF‐α* were analyzed by real‐time PCR. Data are presented as means ± SEM (n = 4). *P < 0.05, **P < 0.01 and ***P < 0.001. **(H)** The concentrations of IL‐1β, IL‐6, IL‐8, TNF‐α and IL-10 in IPEC-J2 cells culture medium were measured by ELISA. Data are presented as means ± SEM (n = 4). *P < 0.05, **P < 0.01 and ***P < 0.001.

To further confirm whether ATRA attenuated TGEV-induced inflammatory response *via* suppressing the TLRs/RLRs/NF‐κB signaling pathway, we used siRNAs targeting specific receptors to interfere with the signaling pathway. The knockdown efficiency of each siRNA was demonstrated by transfection and real‐time PCR assays (**Figure 9A**). The cells were transfected with specific siRNAs followed by infection with TGEV, then incubation with or without ATRA. As shown in **Figures 9B, C**, siRNAs targeting RIG-I and MDA5 but not TLR3 and TLR7 significantly reduced the phosphorylation level of NF-κB p65 in TGEV-infected cells (*P* < 0.01). Next, we further determine the effects of siRNAs on TGEV-induced inflammatory response. As shown in **Figures 9D–H**, knockdown of RIG-I and MDA5 significantly prevented the upregulation of *IL-1β*, *IL-6* and *TNF-α* mRNA abundance and IL-1β, IL-8 and TNF-α concentrations induced by TGEV (*P* < 0.05); knockdown of RIG-I also significantly inhibited the enhancing of IL-10 concentration induced by TGEV (*P* < 0.01); knockdown of MDA5 also significantly inhibited the enhancing of *IL-8* mRNA abundance and IL-6 concentration induced by TGEV (*P* < 0.01); knockdown of TLR3 significantly inhibited the upregulation of *IL-6* mRNA abundance induced by TGEV (*P* < 0.01). However, knockdown of TLR7 had no significant effect on the levels of inflammatory factors in TGEV-infected cells (*P* > 0.05).

## Discussion

Coronaviruses, a genus in the Coronaviridae family, constitute a significant threat to both human and animal health (26, 27). Transmissible gastroenteritis virus (TGEV) is an enteropathogenic coronavirus, which causes severe diarrhea, vomiting and high mortality in piglets, resulting in heavy economic losses to the pig breeding industry (28). TGEV replicates in enterocytes covering the villi of the porcine small intestine, causing villus atrophy and crypt hyperplasia, and impairing the intestinal barrier in diseased piglets (29). The pathogenesis of TGEV infection is strongly associated with inflammation. Previous studies found that the mRNA levels of proinflammatory cytokines *IL-1β*, *IL-6* and *TNF-α* were significantly increased in the jejunum of piglets infected with TGEV (8). Our results also found that the concentrations and mRNA expressions of proinflammatory cytokines *(*IL-1β, IL-6, IL-8 and TNF-α) were significantly increased in TGEV-infected IPEC-J2 cells. However, the up-regulation of anti-inflammatory cytokine IL-10 level also observed in TGEV-infected IPEC-J2 cells. This may be due to the fact that in some circumstances, elevated IL-10 level can induce immunosuppression, allowing the virus to persistence (30). All-trans retinoic acid (ATRA) is an active metabolite of VA, as an immunomodulator, plays important roles in regulating immune response, especially under inflammation conditions (15). Xu et al. reported that ATRA treatment obviously suppressed the up‐regulation of pro-inflammatory cytokines *(IL-1β*, *IL-6*, *IL-17*, and *TNF-α*) mRNA expressions induced by LPS in bovine adipocytes (31). The results indicated that ATRA can inhibit LPS-induced inflammatory response, but it is unclear whether ATRA can attenuate the inflammatory response induced by TGEV. In the present study, we found that ATRA treatment reduced the up‐regulation of inflammatory cytokines (*IL-1β*, *IL-6*, *IL-8*, *TNF-α* and *IL-10*) mRNA expressions induced by TGEV in a dose-dependent manner. To further determine the effects of ATRA on the TGEV‐induced inflammatory response, we detected the concentrations of inflammatory cytokines in cell culture medium. Our results found that ATRA treatment significantly inhibited the elevation of IL-1β, IL-6, TNF-α and IL-10 concentrations induced by TGEV. These results indicated that ATRA can attenuate TGEV-induced inflammatory response in IPEC-J2 cells.

The function of epithelial cells is based on the integrity of the epithelial barrier, and excessive production of proinflammatory cytokines might lead to the cell damage (32). LDH is an important indicator of the intestinal epithelial barrier integrity. It is known that LDH is released when the cells are damaged, so LDH release can indirectly reflect the permeability of cells (33). In the present study, TGEV infection significantly increased the LDH activity in IPEC-J2 cells culture medium, whereas ATRA treatment significantly decreased LDH released from the TGEV-infected IPEC-J2 cells. These results suggested that the ATRA treatment prevented the TGEV-induced enhancement of cell permeability. Tight junction (TJ) proteins [occludin, claudin, and intracellular plaque proteins (ZO and cingluin)] play important roles in maintaining the mechanical barrier and permeability of the mucosal epithelium (34). Studies have shown that proinflammatory cytokines (IL-1β, IL-6, IL-8 and TNF-α) could induce disruption of tight junction proteins, which led to the increasing of intestinal permeability (35). In the present study, consistent with overproduction of proinflammatory cytokines induced by TGEV, decreased ZO-1 and Occludin protein levels in TGEV-infected IPEC-J2 cells were observed. However, ATRA treatment significantly reversed the reduction of ZO-1 and Occludin protein levels induced by TGEV. Similar results also reported that ATRA treatment significantly suppressed the downregulation of ZO-1, Occludin and Claudin-1 protein levels induced by LPS in IPEC-J2 (18). These results indicated that ATRA may attenuate TGEV-induced epithelial barrier integrity damage in IPEC-J2 cells via suppressing inflammatory response.

To further study the mechanism by which ATRA suppress TGEV-induced inflammatory response in IPEC-J2 cells, we detected NF-κB signaling pathway. NF‐κB is a major transcription factor that plays an essential role in inflammatory response. In the stimulated state, IκBα gets phosphorylated and dissociated from NF‐κB, and then NF‐κB gets phosphorylated and migrates into the nucleus to promote the release of pro-inflammatory cytokines (36, 37). Wang et al. reported that TGEV infection promoted the phosphorylation of NF-κB p65 and nuclear translocation of NF‐κB p65, and leading to an increase in pro-inflammatory cytokines mRNA expressions in IPEC-J2 and ST cells (9). In the present study, TGEV infection significantly upregulated the phosphorylation levels of IκBα and NF-κB p65, and induced the translocation of NF-κB p65 from cytoplasm to nucleus in IPEC-J2 cells, while ATRA treatment inhibited this process. These results indicated that ATRA can inhibit TGEV-induced NF-κB signaling pathway activation. To further investigate whether ATRA alleviated TGEV-induced inflammatory response via suppressing the NF‐κB signaling pathway, we used the NF-κB specific inhibitors (BAY11-7082) to inhibit NF-κB activation in TGEV-infected IPEC-J2 cells. Our results found that BAY11-7082 treatment significantly inhibited the enhancing of NF-κB p65 phosphorylation level induced by TGEV. Furthermore, we found that BAY11-7082 treatment significantly inhibited the upregulation of pro-inflammatory cytokines (IL-1β, IL-6, IL-8 or TNF-α) levels induced by TGEV. These results indicated that ATRA may attenuate TGEV-induced inflammatory response via suppressing the NF‐κB signaling pathway. However, the underlying mechanisms of how ATRA negatively regulated the NF‐κB signaling pathway remain unclear.

At present, Toll-like receptors (TLRs), and RIG-I-like receptors (RLRs) are the two main pattern recognition receptors (PRRs) for detecting viral pathogen-associated molecular patterns (PAMPs) (38). After the virus recognizes PRRs, these receptors recruit specific intracellular adaptor proteins (MyD88, TRIF or MAVS) to trigger downstream signaling events, thereby activating NF-κB and inducing the production of inflammatory cytokines (39, 40). Previous studies have shown PEDV virus and herpesvirus can induce NF-κB activation by the TLRs-mediated signaling pathway (41, 42). In the present study, TGEV infection significantly upregulated the mRNA levels of TLR2, TLR3, TLR4, TLR7 and their downstream signaling molecules MyD88, TRIF and TRAF6. However, ATRA treatment significantly suppressed the upregulation of TLR3, TLR7, TRIF and TRAF6 mRNA levels induced by TGEV. Furthermore, we found that ATRA treatment significantly inhibited the enhancing of TLR3 and TLR7 protein levels induced by TGEV. These results indicated that ATRA may suppress the TLR3 and TLR7 signaling pathway in TGEV-infected IPEC-J2 cells. Moreover, Zhen et al. reported that TGEV infection induces NF-κB activation through RLRs-mediated signaling pathway (43). In the present study, we also found that TGEV infection significantly increased the mRNA abundance of RIG-I, MDA5 and MAVS. However, ATRA treatment significantly inhibited the enhancing of RIG-I, MDA5 and MAVS mRNA abundance induced by TGEV. We further researched and found that ATRA treatment significantly inhibited the enhancing of RIG-I and MDA5 protein levels induced by TGEV. These results indicated that ATRA may suppress the RLRs signaling pathway in TGEV-infected IPEC-J2 cells. To further confirm whether ATRA alleviated TGEV-induced inflammatory response via suppressing the TLRs/RLRs/NF‐κB signaling pathway, we used siRNAs targeting specific receptors to interfere with the signaling pathways. Our studies showed that TLR3 and TLR7 knockdown did not impede TGEV-induced NF-κB activation; however, RIG-I and MDA5 knockdown clearly suppressed TGEV-induced NF-κB activation. These results indicated that ATRA attenuated TGEV-induced NF-κB activation *via* suppressing the RLRs signaling pathway. Next, we further determine the effects of siRNAs on TGEV-induced inflammatory response. Our results found that siRNAs targeting RIG-I and MDA5 but not TLR3 and TLR7 significantly reduced the mRNA expressions and concentrations of pro-inflammatory cytokines (IL-1β, IL-6, IL-8 or TNF-α) in TGEV-infected cells. The above results suggested that ATRA alleviated TGEV-induced inflammatory response *via* suppressing the RLRs/NF‐κB signaling pathway.

**Figure 10:**
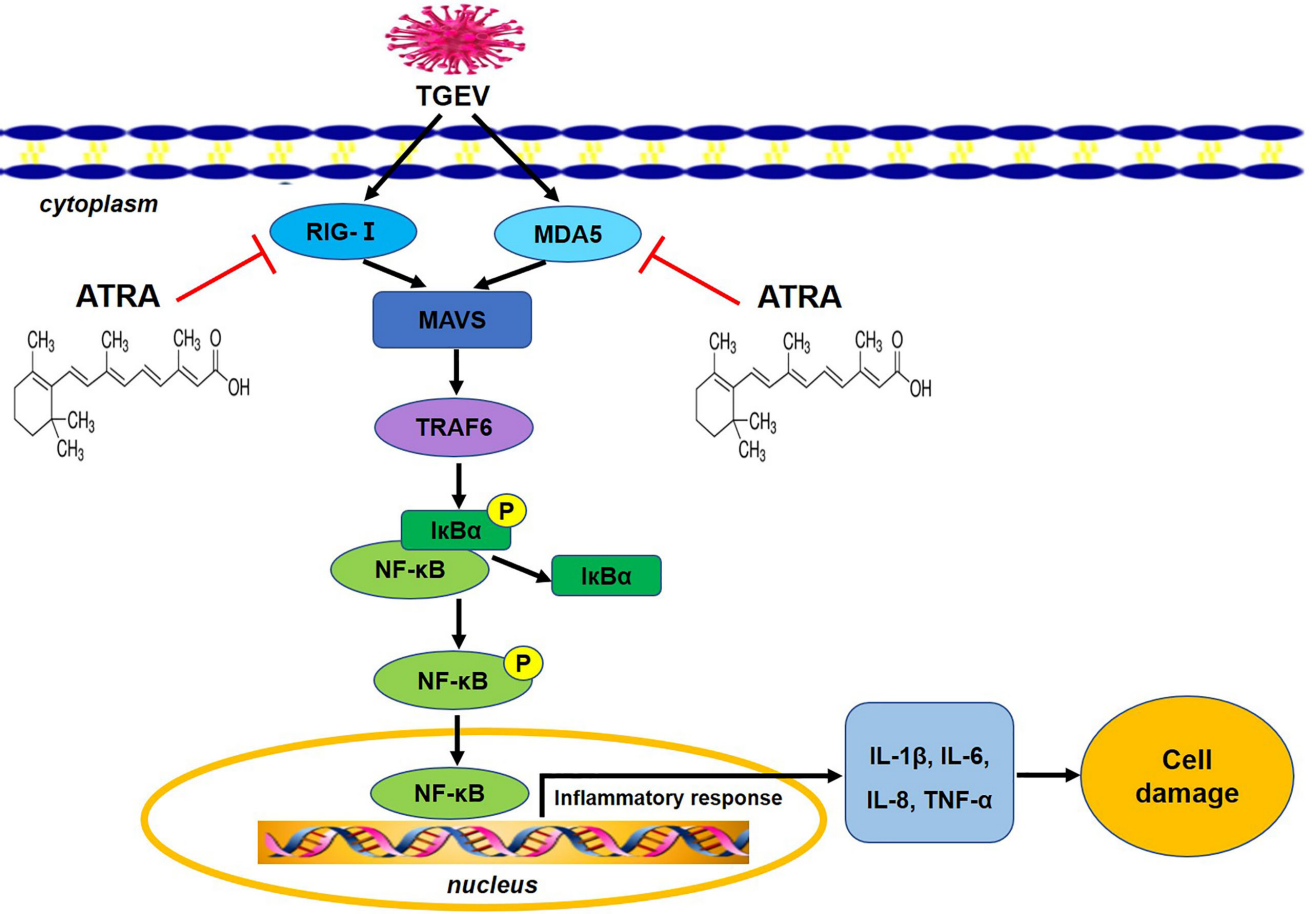
The potential mechanism of ATRA attenuated TGEV-induced inflammatory injury in IPEC-J2 cells. ATRA attenuated TGEV-induced inflammatory injury in IPEC-J2 cells *via* suppressing the RLRs/NF‐κB signaling pathway.

In conclusion, our results indicated that ATRA attenuated TGEV-induced IPEC-J2 cells damage *via* suppressing inflammatory response, the mechanism of which is associated with the inhibition of TGEV-mediated activation of RLRs/NF‐κB signaling pathway **Figure 10**. These results provide a scientific basis for considering ATRA as a potential therapeutic agent against TGEV infection in the future.

## Data Availability Statement

The original contributions presented in the study are included in the article/**Supplementary Material**. Further inquiries can be directed to the corresponding author.

## Author Contributions

BY, JP, and DC conceived and designed the experiments. JP, GT, JH, ZH, PZ, XM, JY, JL, YL, and HY performed the experiments and data analysis. JP wrote the manuscript. BY and DC revised the manuscript. All authors contributed to the article and approved the submitted version.

## Funding

The present study was supported by the National Key Research and Development Program of China (2018YFD0500605) and the Sichuan Pig Innovation Team of National Modern Agricultural Industry Technology System of China (scsztd-2020-08-11).

## Conflict of Interest

The authors declare that the research was conducted in the absence of any commercial or financial relationships that could be construed as a potential conflict of interest.

## Publisher’s Note

All claims expressed in this article are solely those of the authors and do not necessarily represent those of their affiliated organizations, or those of the publisher, the editors and the reviewers. Any product that may be evaluated in this article, or claim that may be made by its manufacturer, is not guaranteed or endorsed by the publisher.
