# Supplementary figures and images for: All-Trans Retinoic Acid Attenuates Transmissible Gastroenteritis Virus-Induced Inflammation in IPEC-J2 Cells via Suppressing the RLRs/NF‐κB Signaling Pathway
Source: Front Immunol. 2022 Jan 31;13:734171. doi: 10.3389/fimmu.2022.734171 (PMC8841732; doi:10.3389/fimmu.2022.734171)

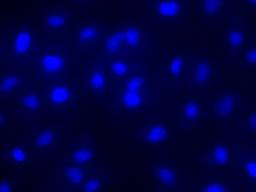

Supplement: Supplementary file 1 [file DataSheet_1.zip › Raw data-2021-12-18/Raw data-Immunofluorescence/ATRA/.Metadata/1_image_DAP.tif.thb]

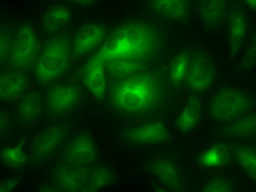

Supplement: Supplementary file 1 [file DataSheet_1.zip › Raw data-2021-12-18/Raw data-Immunofluorescence/ATRA/.Metadata/2_image_L5.tif.thb]

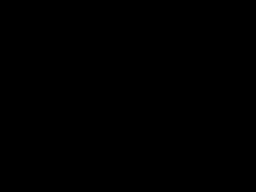

Supplement: Supplementary file 1 [file DataSheet_1.zip › Raw data-2021-12-18/Raw data-Immunofluorescence/ATRA/.Metadata/3_image_Y3.tif.thb]

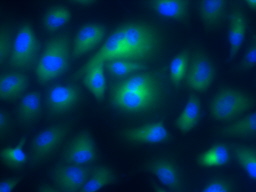

Supplement: Supplementary file 1 [file DataSheet_1.zip › Raw data-2021-12-18/Raw data-Immunofluorescence/ATRA/.Metadata/Overlay_Maximum.tif.thb]

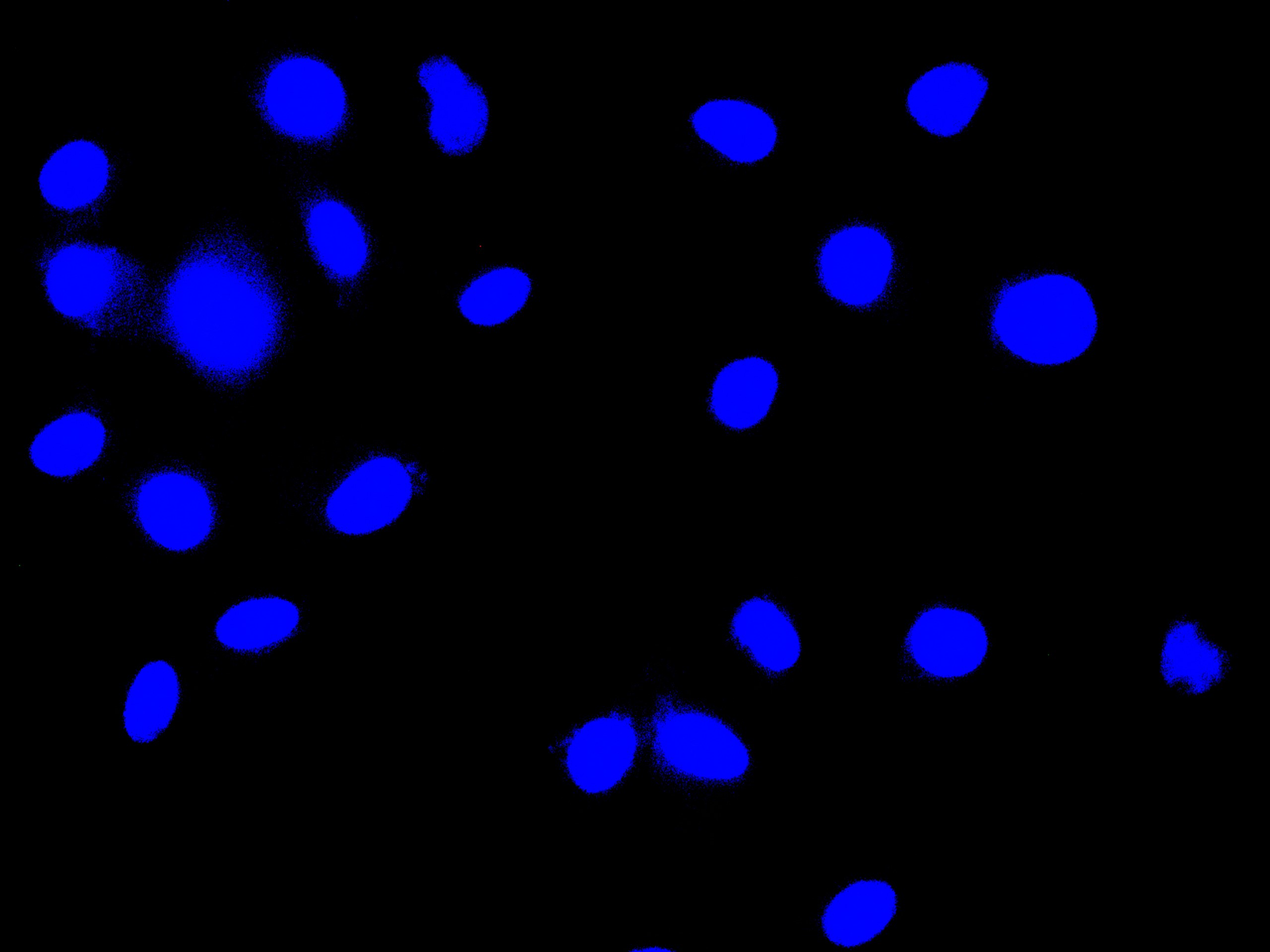

Supplement: Supplementary file 1 [file DataSheet_1.zip › Raw data-2021-12-18/Raw data-Immunofluorescence/ATRA/1_image_DAP-1.jpg]

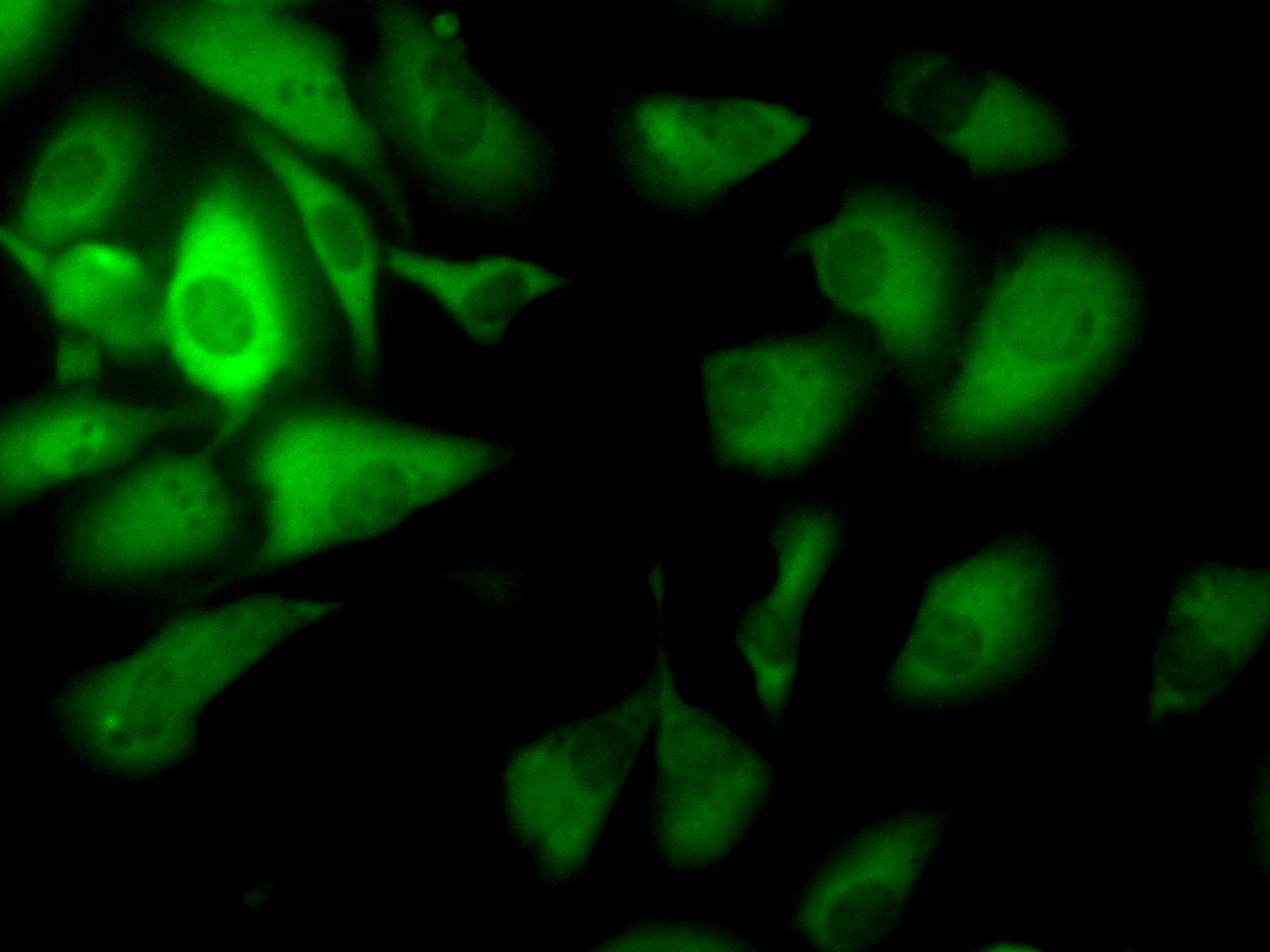

Supplement: Supplementary file 1 [file DataSheet_1.zip › Raw data-2021-12-18/Raw data-Immunofluorescence/ATRA/2_image_L5-1.jpg]

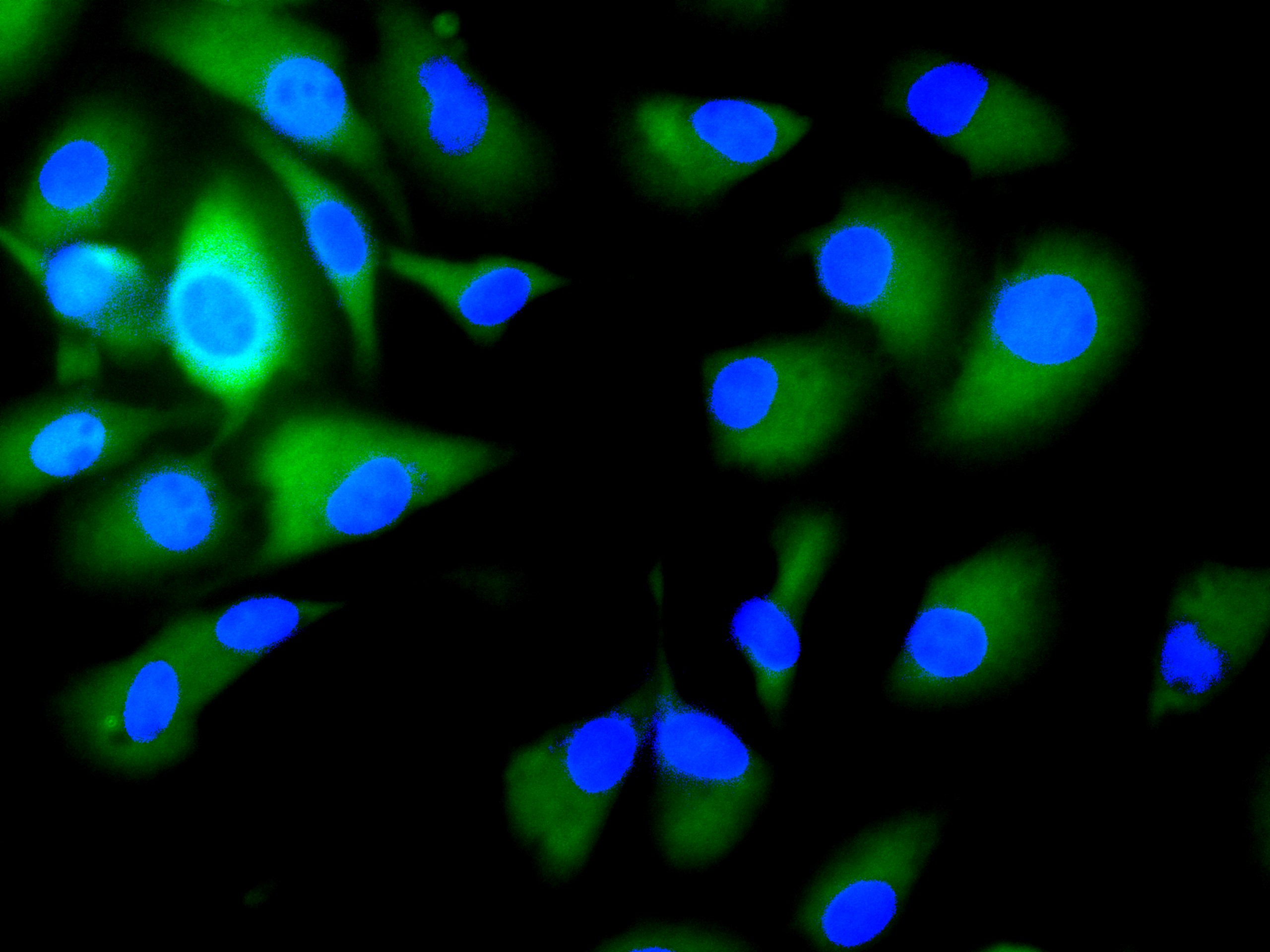

Supplement: Supplementary file 1 [file DataSheet_1.zip › Raw data-2021-12-18/Raw data-Immunofluorescence/ATRA/Overlay_Maximum.jpg]

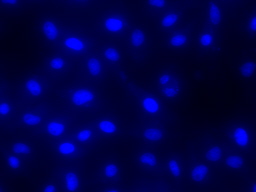

Supplement: Supplementary file 1 [file DataSheet_1.zip › Raw data-2021-12-18/Raw data-Immunofluorescence/Control/.Metadata/1_image_DAP.tif.thb]

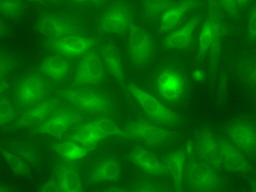

Supplement: Supplementary file 1 [file DataSheet_1.zip › Raw data-2021-12-18/Raw data-Immunofluorescence/Control/.Metadata/2_image_L5.tif.thb]

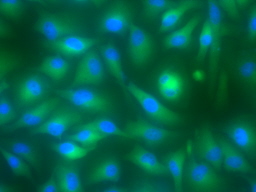

Supplement: Supplementary file 1 [file DataSheet_1.zip › Raw data-2021-12-18/Raw data-Immunofluorescence/Control/.Metadata/Overlay_Maximum.tif.thb]

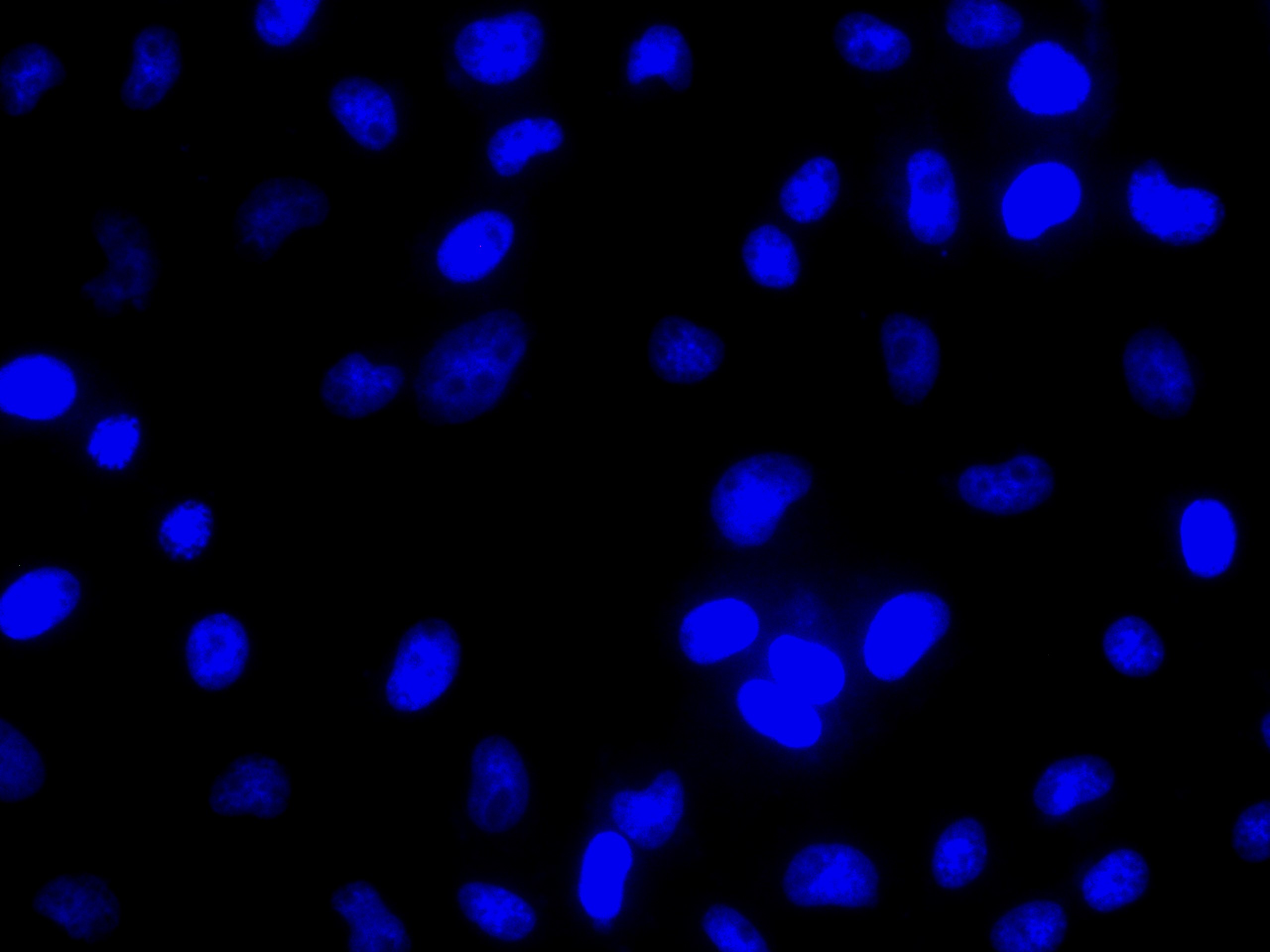

Supplement: Supplementary file 1 [file DataSheet_1.zip › Raw data-2021-12-18/Raw data-Immunofluorescence/Control/1_image_DAP-1.jpg]

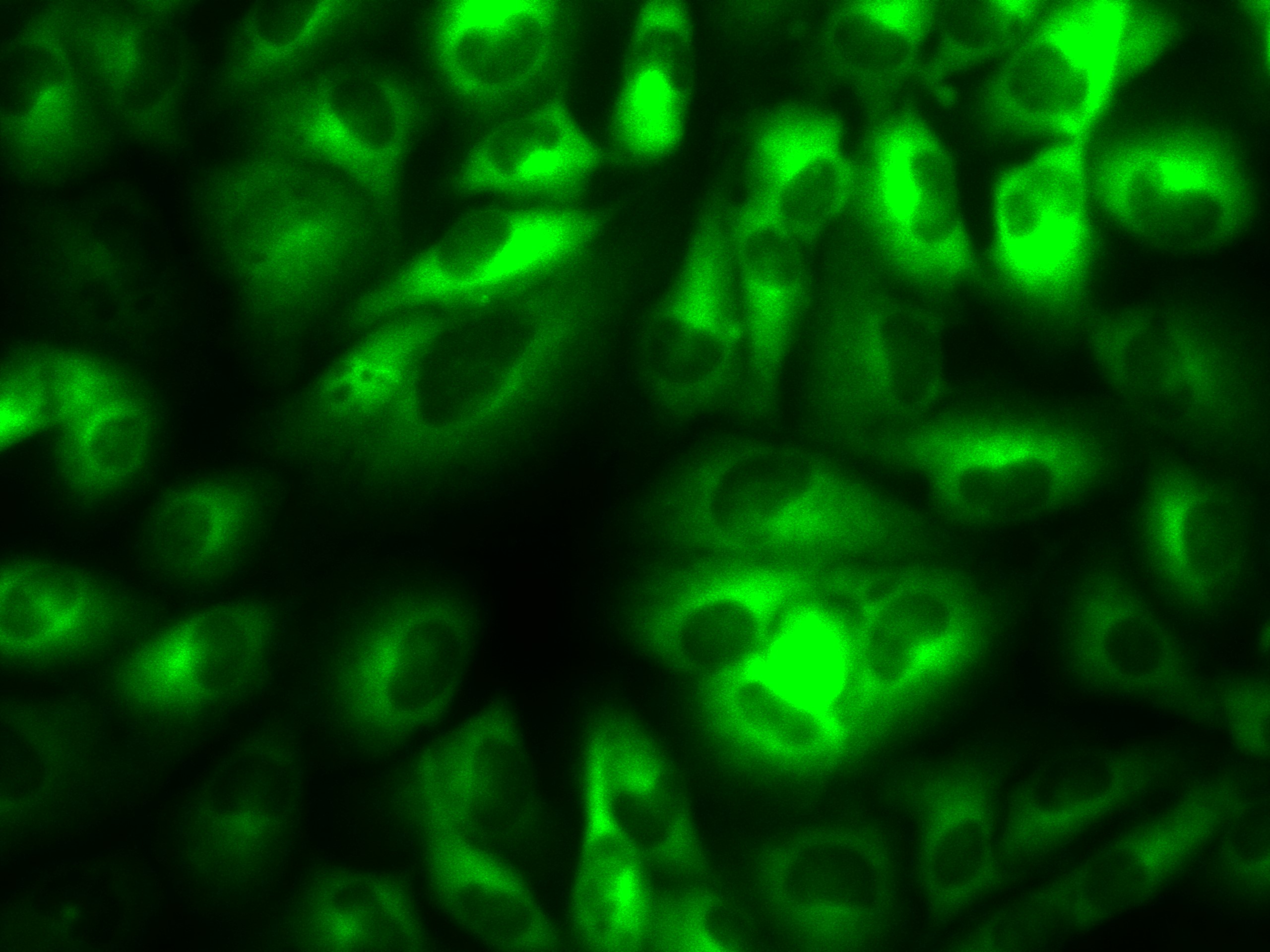

Supplement: Supplementary file 1 [file DataSheet_1.zip › Raw data-2021-12-18/Raw data-Immunofluorescence/Control/2_image_L5-1.jpg]

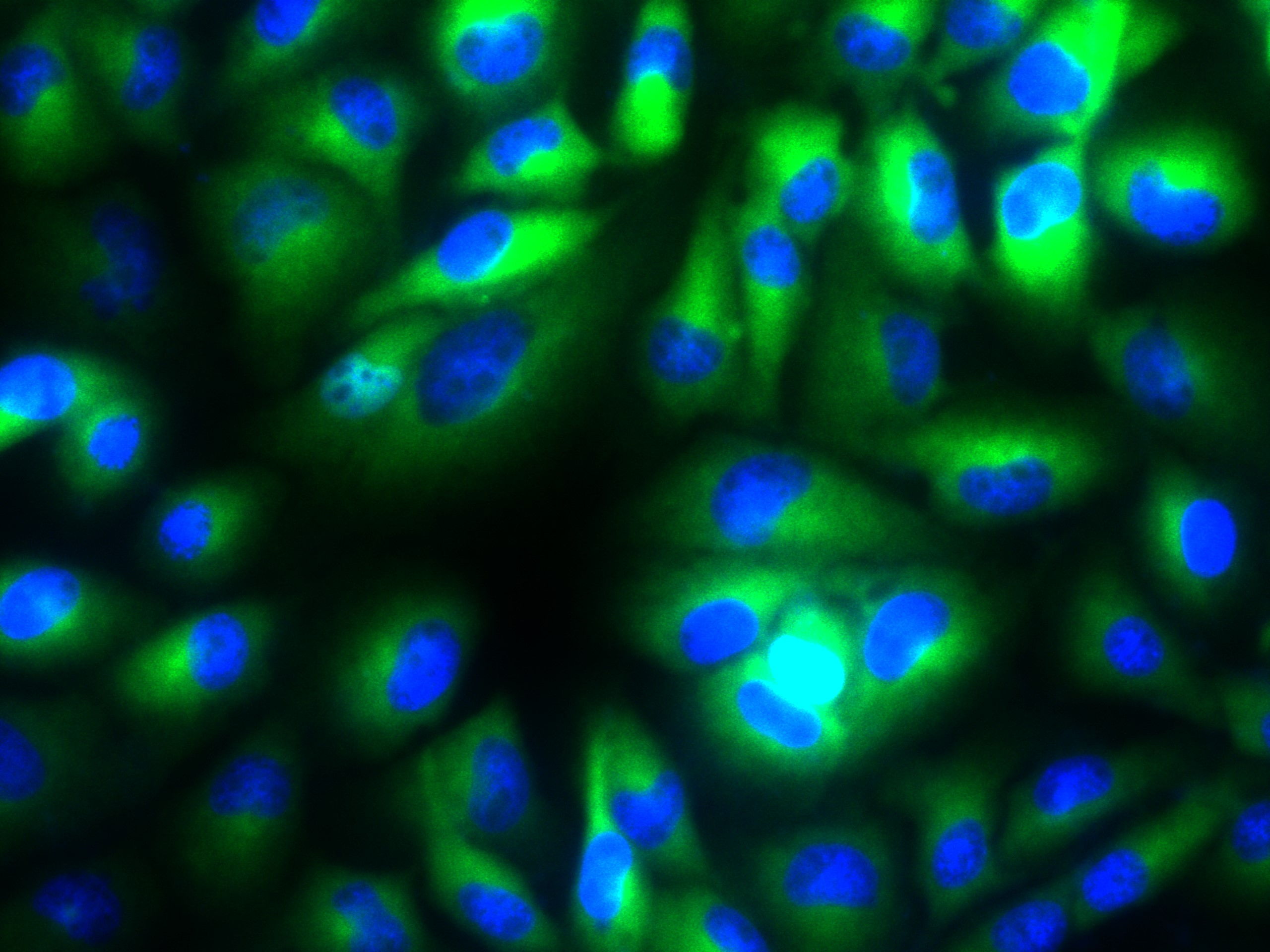

Supplement: Supplementary file 1 [file DataSheet_1.zip › Raw data-2021-12-18/Raw data-Immunofluorescence/Control/Overlay_Maximum-1.jpg]

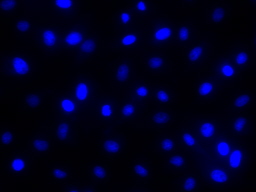

Supplement: Supplementary file 1 [file DataSheet_1.zip › Raw data-2021-12-18/Raw data-Immunofluorescence/T+ATRA/.Metadata/1_image_DAP.tif.thb]

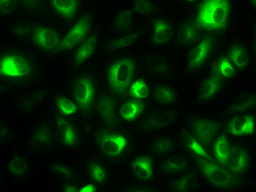

Supplement: Supplementary file 1 [file DataSheet_1.zip › Raw data-2021-12-18/Raw data-Immunofluorescence/T+ATRA/.Metadata/2_image_L5.tif.thb]

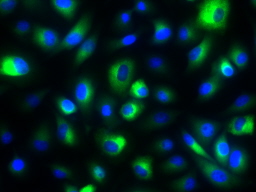

Supplement: Supplementary file 1 [file DataSheet_1.zip › Raw data-2021-12-18/Raw data-Immunofluorescence/T+ATRA/.Metadata/Overlay_Maximum.tif.thb]

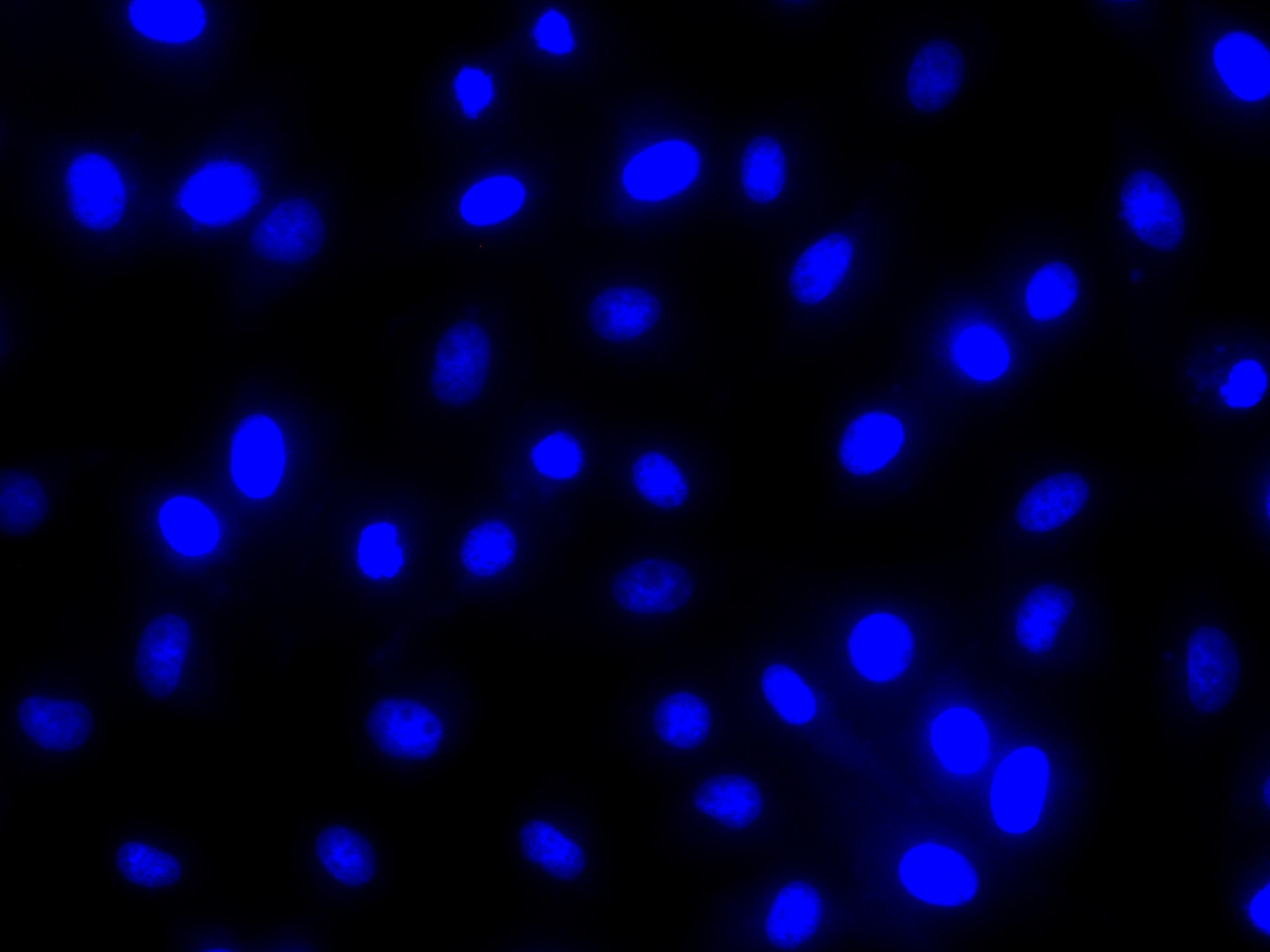

Supplement: Supplementary file 1 [file DataSheet_1.zip › Raw data-2021-12-18/Raw data-Immunofluorescence/T+ATRA/1_image_DAP-1.jpg]

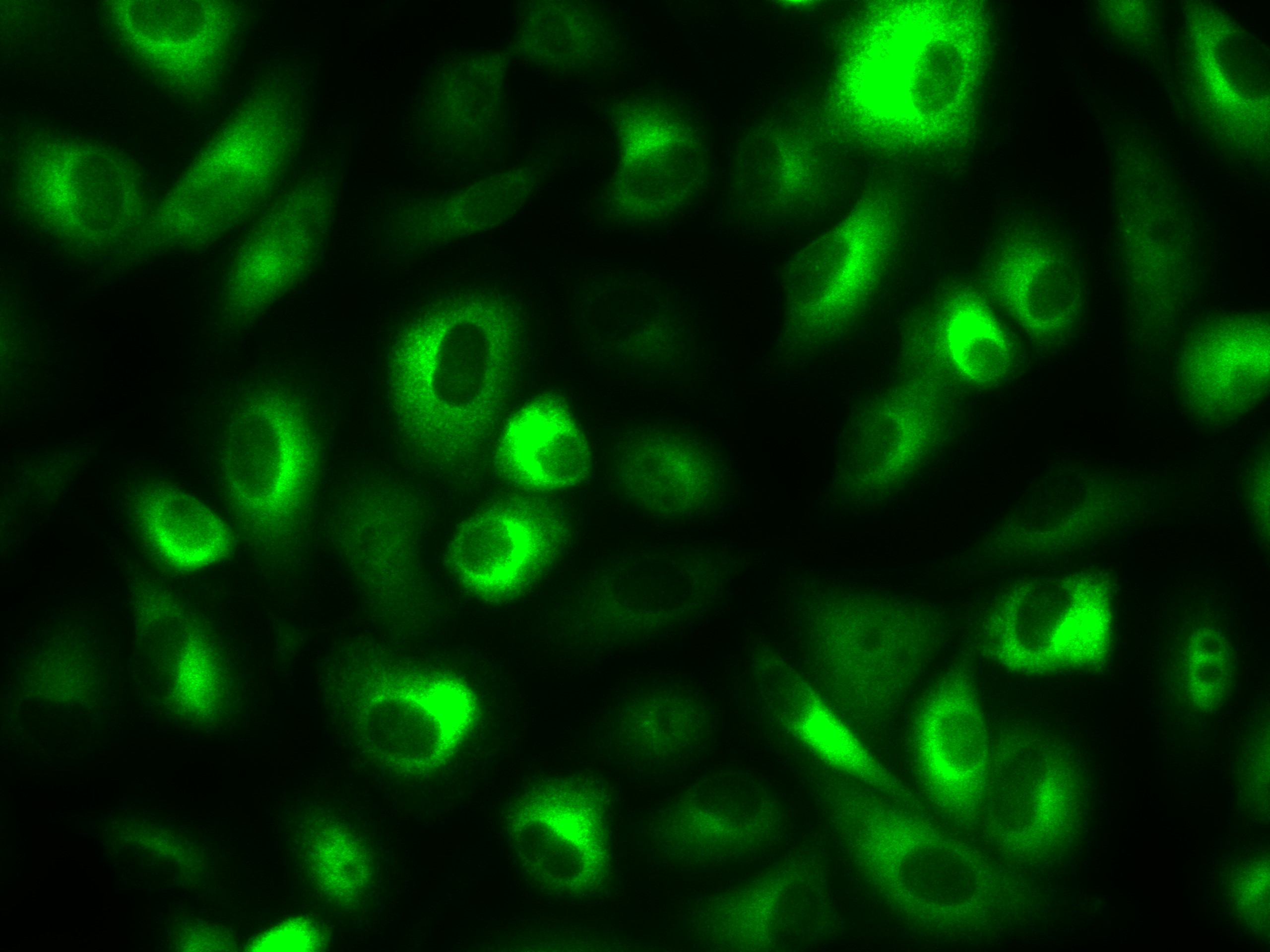

Supplement: Supplementary file 1 [file DataSheet_1.zip › Raw data-2021-12-18/Raw data-Immunofluorescence/T+ATRA/2_image_L5.jpg]

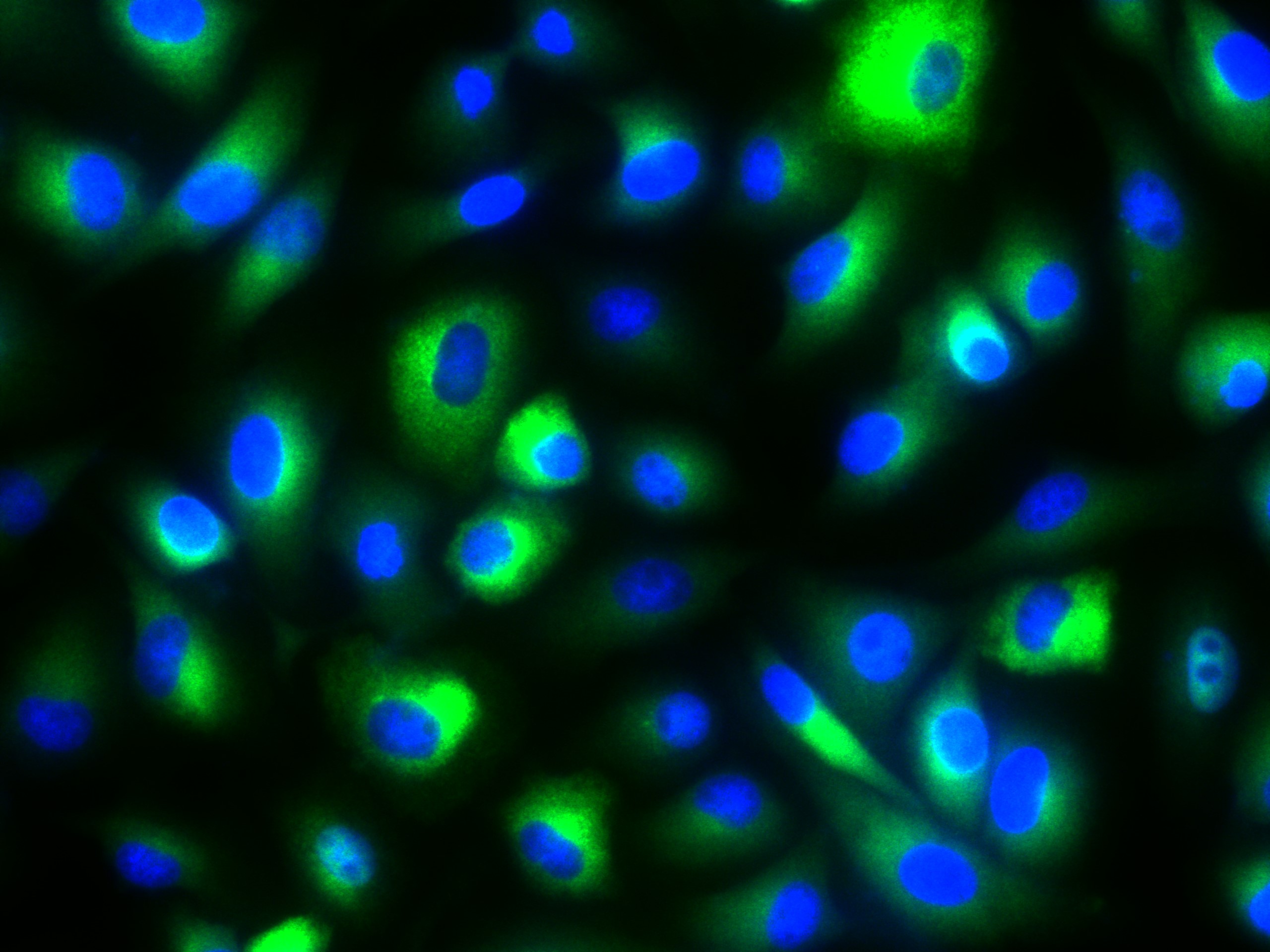

Supplement: Supplementary file 1 [file DataSheet_1.zip › Raw data-2021-12-18/Raw data-Immunofluorescence/T+ATRA/Overlay_Maximum.jpg]

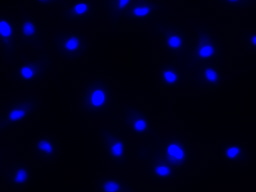

Supplement: Supplementary file 1 [file DataSheet_1.zip › Raw data-2021-12-18/Raw data-Immunofluorescence/TGEV/.Metadata/1_image_DAP.tif.thb]

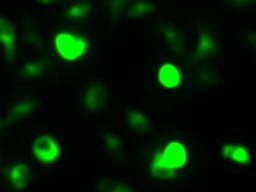

Supplement: Supplementary file 1 [file DataSheet_1.zip › Raw data-2021-12-18/Raw data-Immunofluorescence/TGEV/.Metadata/2_image_L5.tif.thb]

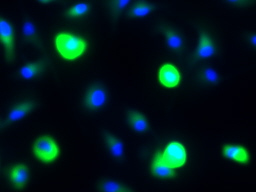

Supplement: Supplementary file 1 [file DataSheet_1.zip › Raw data-2021-12-18/Raw data-Immunofluorescence/TGEV/.Metadata/Overlay_Maximum.tif.thb]

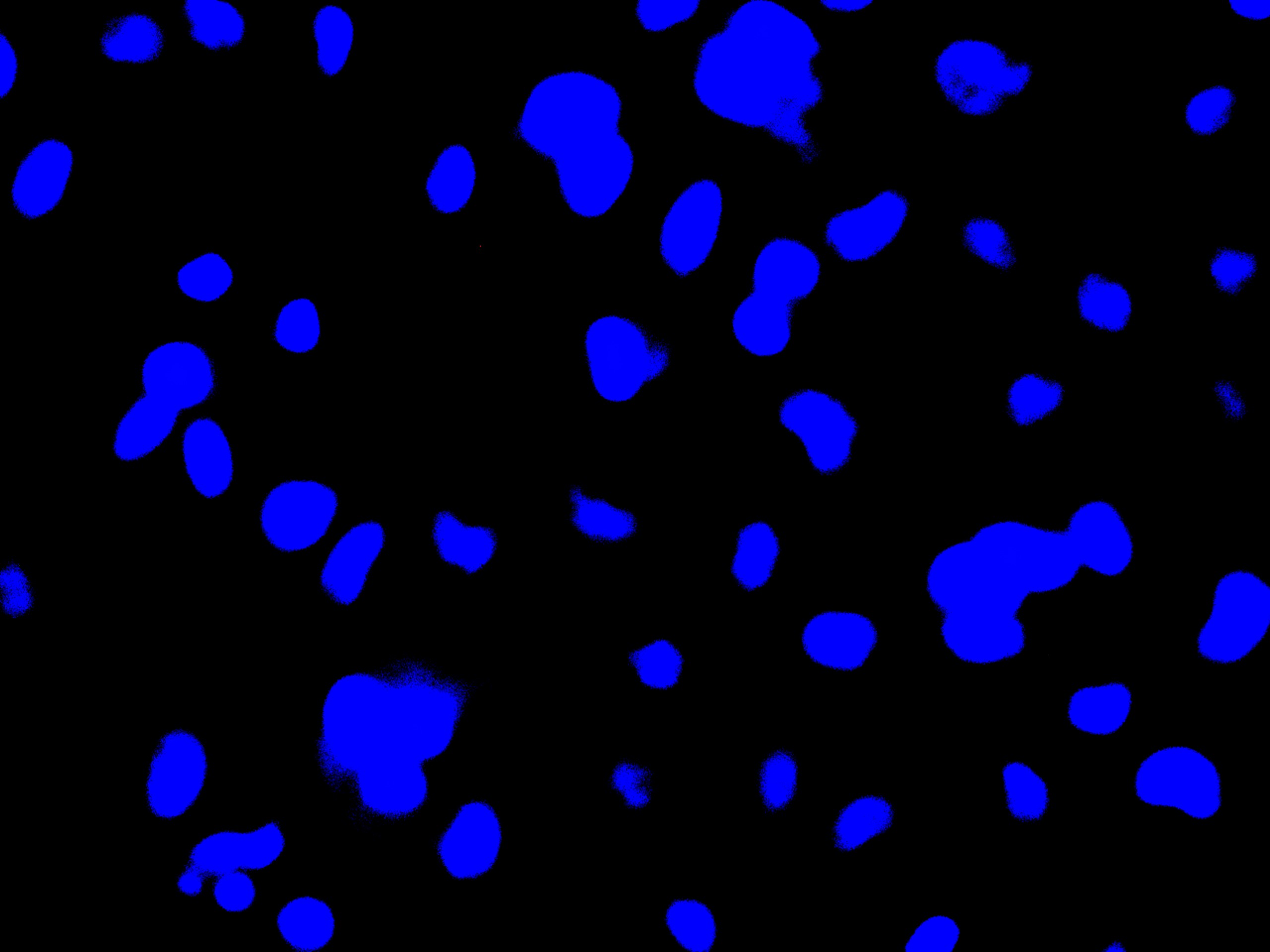

Supplement: Supplementary file 1 [file DataSheet_1.zip › Raw data-2021-12-18/Raw data-Immunofluorescence/TGEV/1_image_DAP-1.jpg]

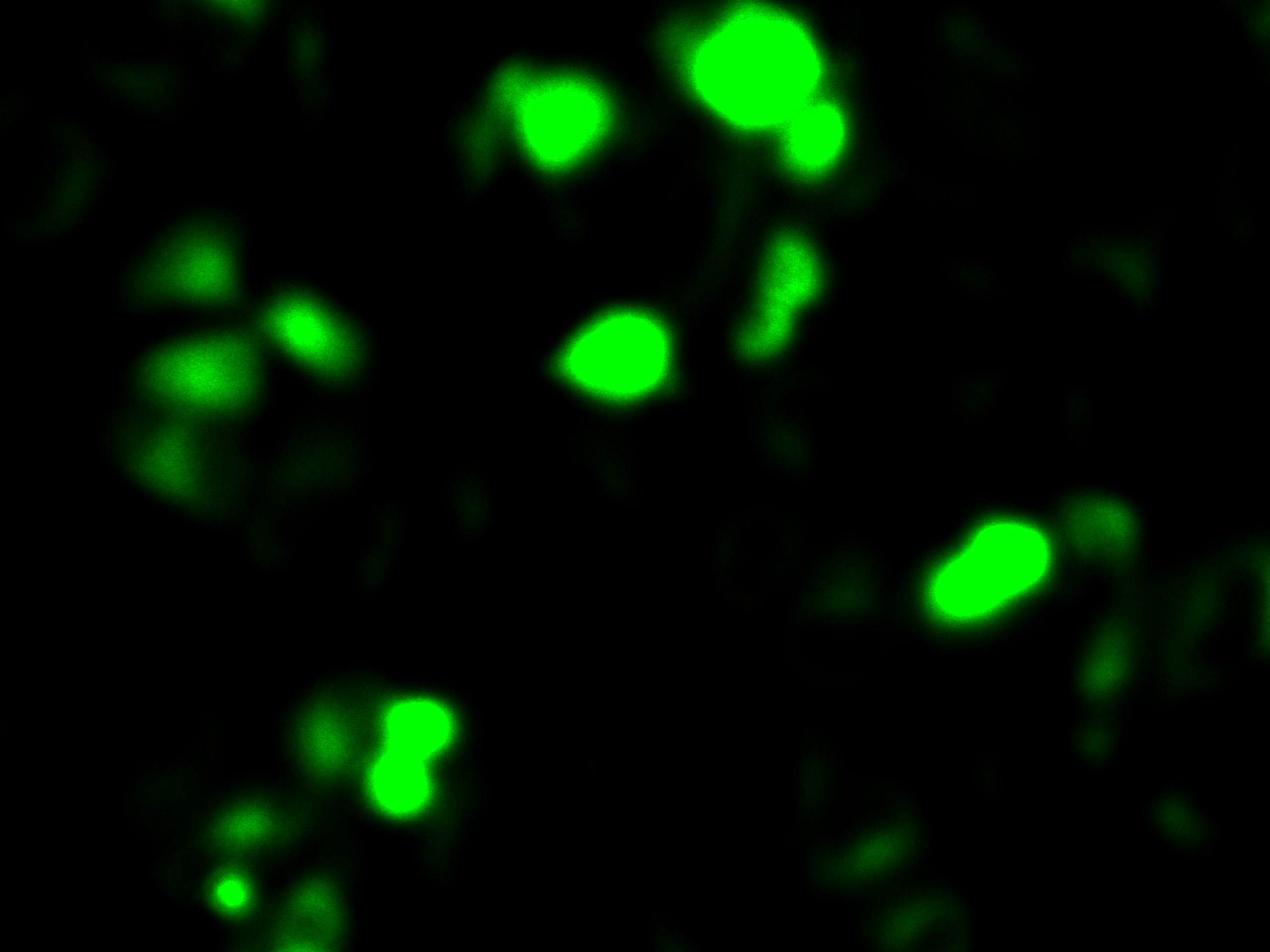

Supplement: Supplementary file 1 [file DataSheet_1.zip › Raw data-2021-12-18/Raw data-Immunofluorescence/TGEV/2_image_L5-1.jpg]

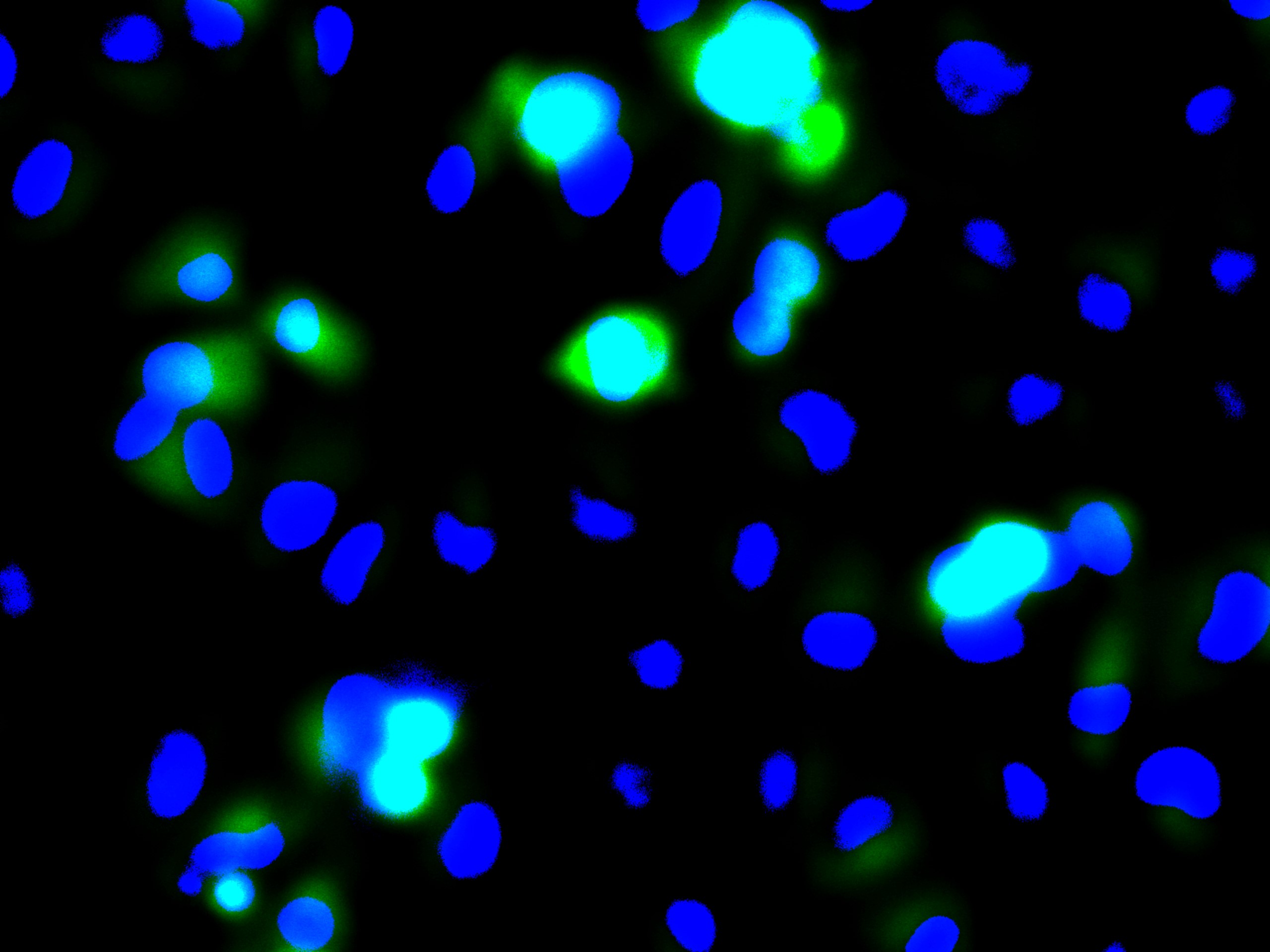

Supplement: Supplementary file 1 [file DataSheet_1.zip › Raw data-2021-12-18/Raw data-Immunofluorescence/TGEV/Overlay_Maximum.jpg]

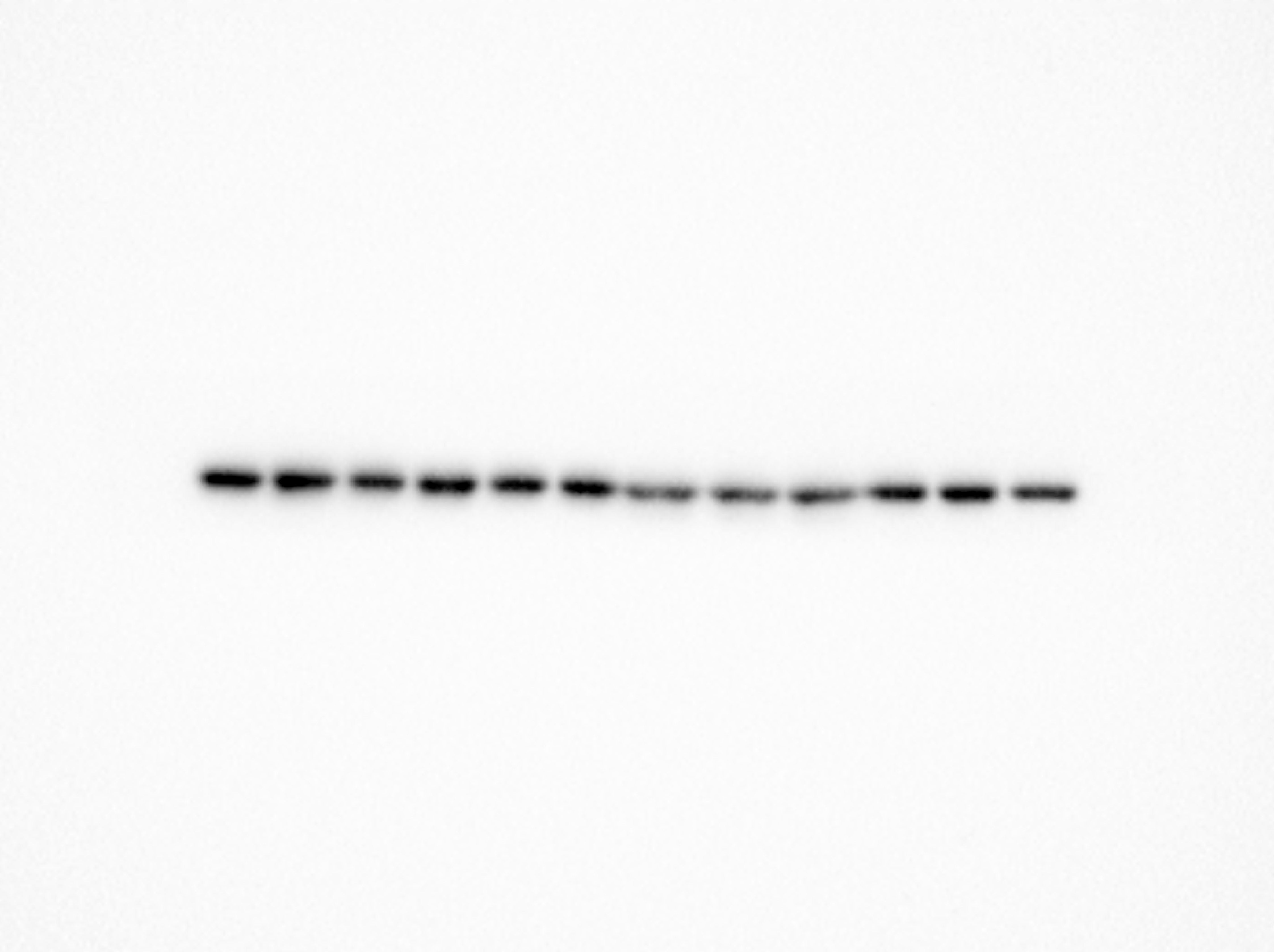

Supplement: Supplementary file 1 [file DataSheet_1.zip › Raw data-2021-12-18/Raw data-Western Blot-2021-12-18/CONíóATRAíóTGEVíóT+ATRA/NF-a╩B signaling pathway/Io▄Ba┴.jpg]

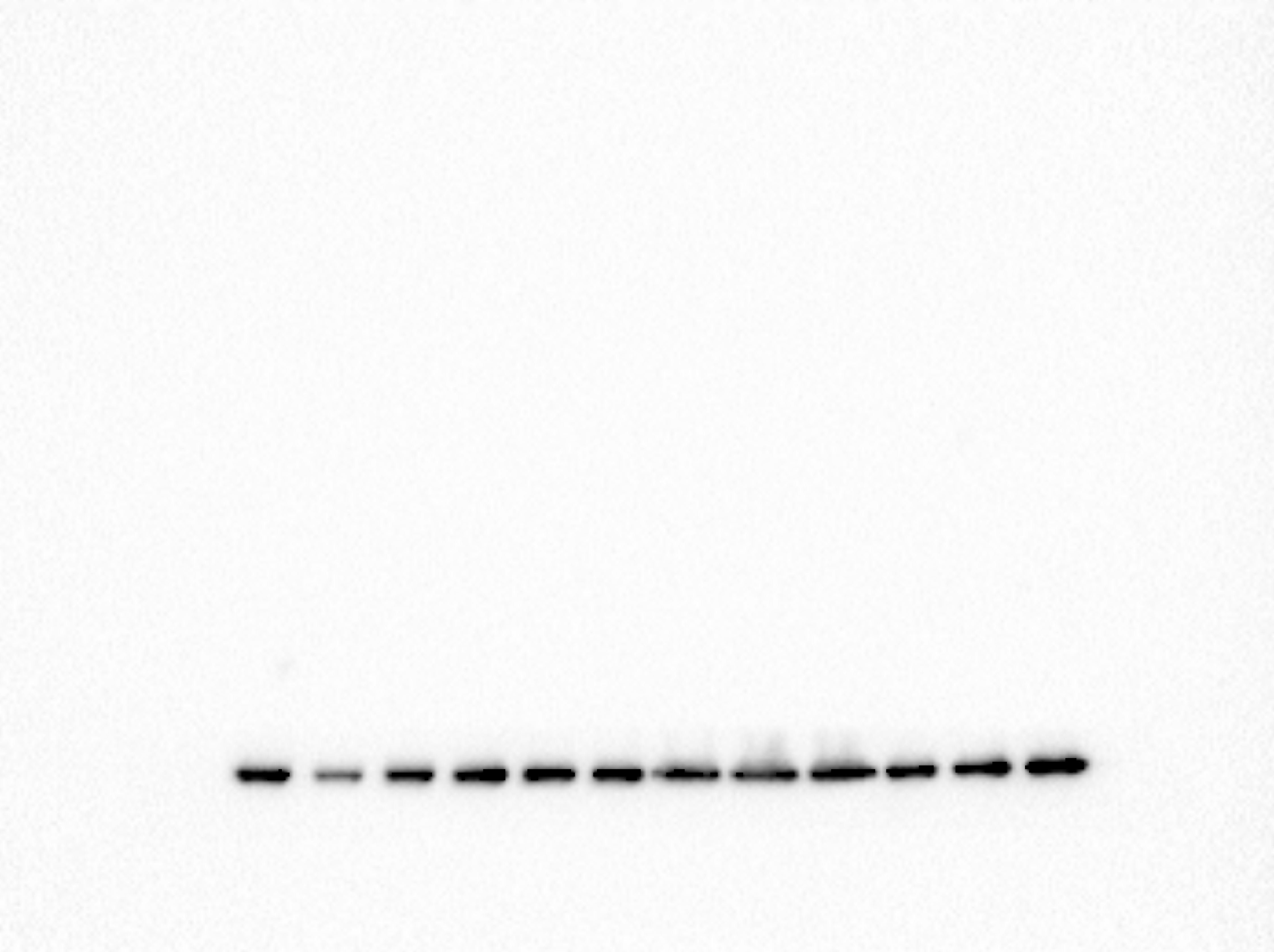

Supplement: Supplementary file 1 [file DataSheet_1.zip › Raw data-2021-12-18/Raw data-Western Blot-2021-12-18/CONíóATRAíóTGEVíóT+ATRA/NF-a╩B signaling pathway/NF-a╩B p65.jpg]

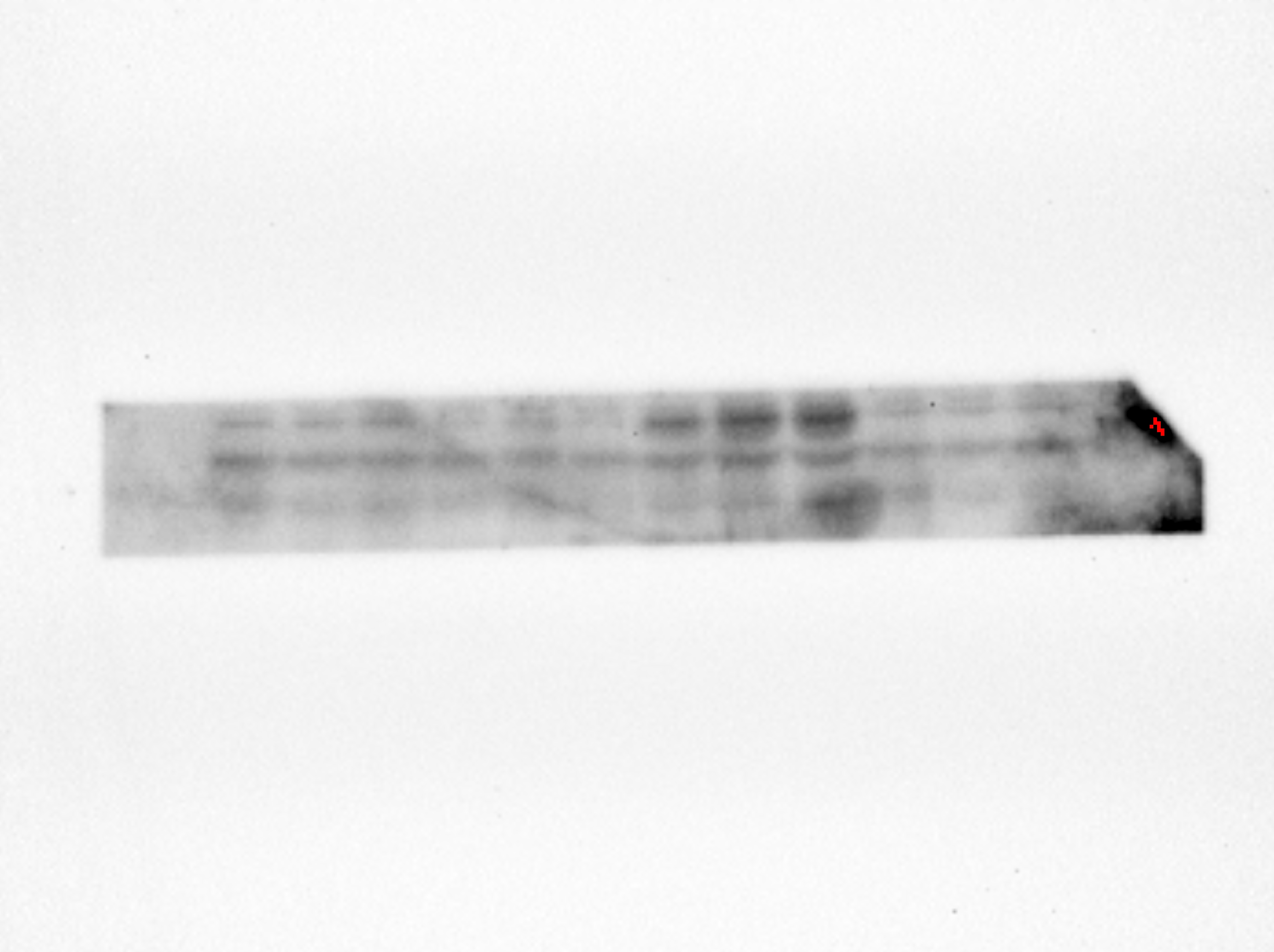

Supplement: Supplementary file 1 [file DataSheet_1.zip › Raw data-2021-12-18/Raw data-Western Blot-2021-12-18/CONíóATRAíóTGEVíóT+ATRA/NF-a╩B signaling pathway/p-Io▄Ba┴.jpg]

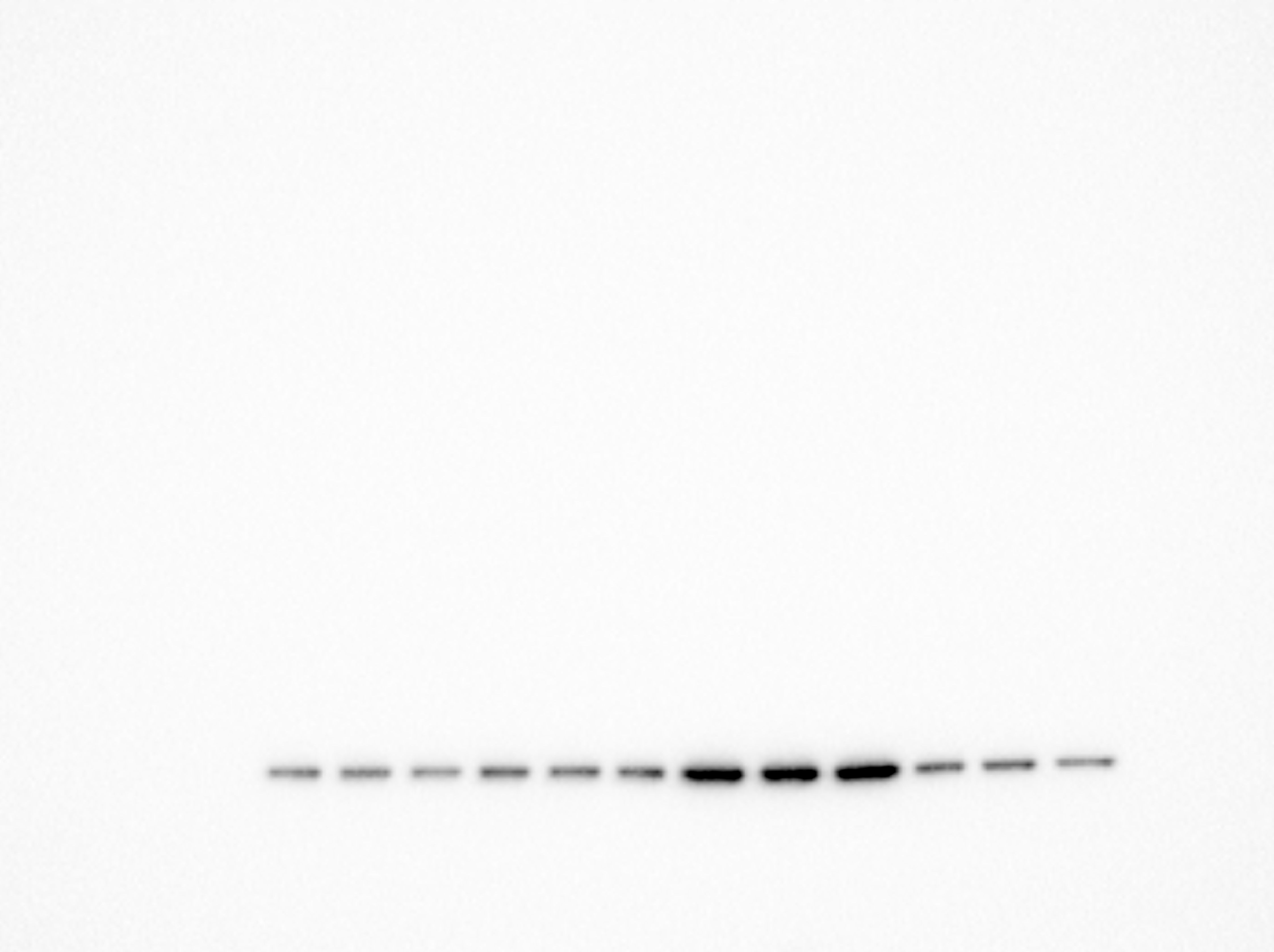

Supplement: Supplementary file 1 [file DataSheet_1.zip › Raw data-2021-12-18/Raw data-Western Blot-2021-12-18/CONíóATRAíóTGEVíóT+ATRA/NF-a╩B signaling pathway/p-NF-a╩B p65.jpg]

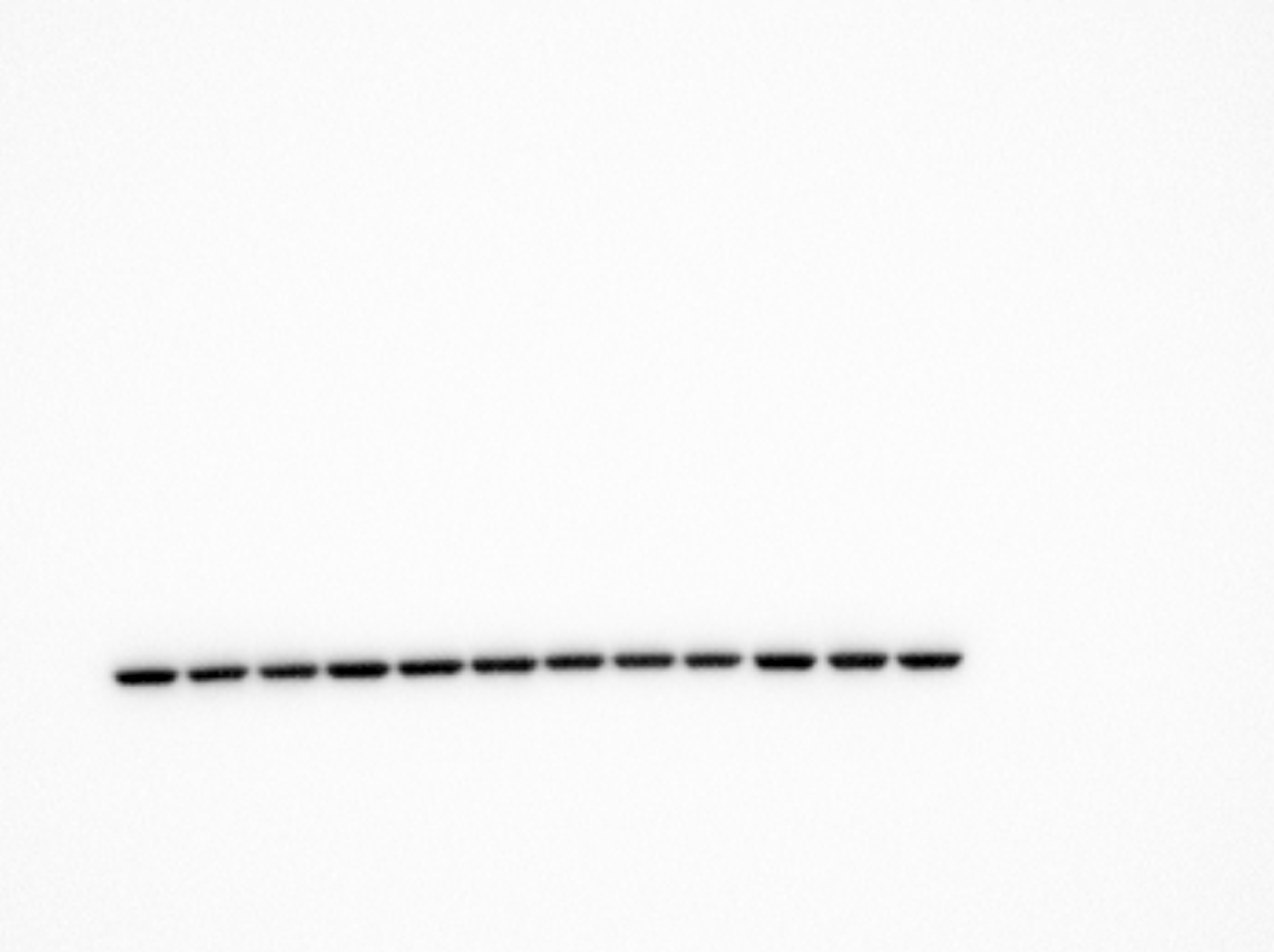

Supplement: Supplementary file 1 [file DataSheet_1.zip › Raw data-2021-12-18/Raw data-Western Blot-2021-12-18/CONíóATRAíóTGEVíóT+ATRA/NF-a╩B signaling pathway/a┬-actin.jpg]

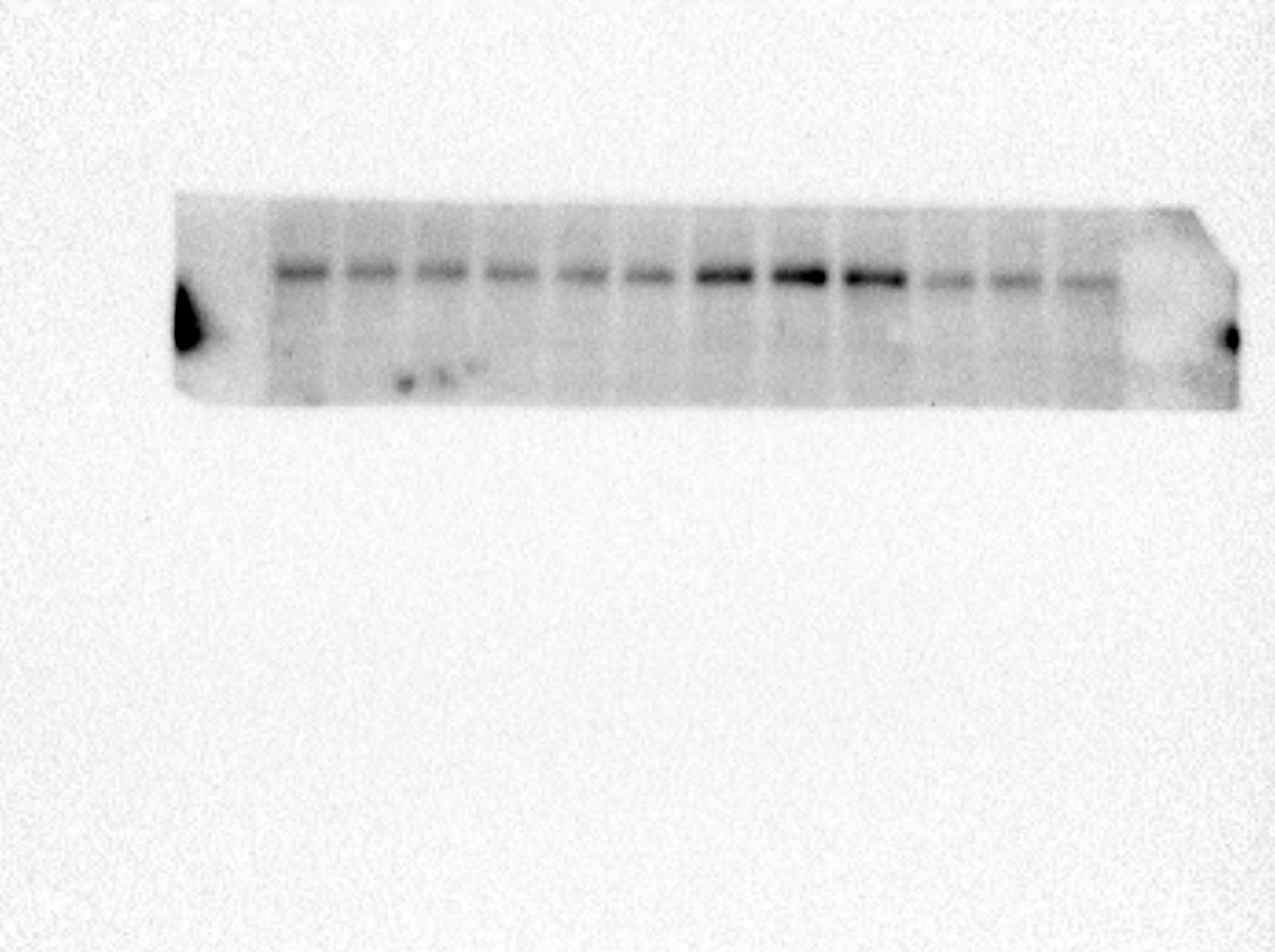

Supplement: Supplementary file 1 [file DataSheet_1.zip › Raw data-2021-12-18/Raw data-Western Blot-2021-12-18/CONíóATRAíóTGEVíóT+ATRA/RLRs/MDA5.jpg]

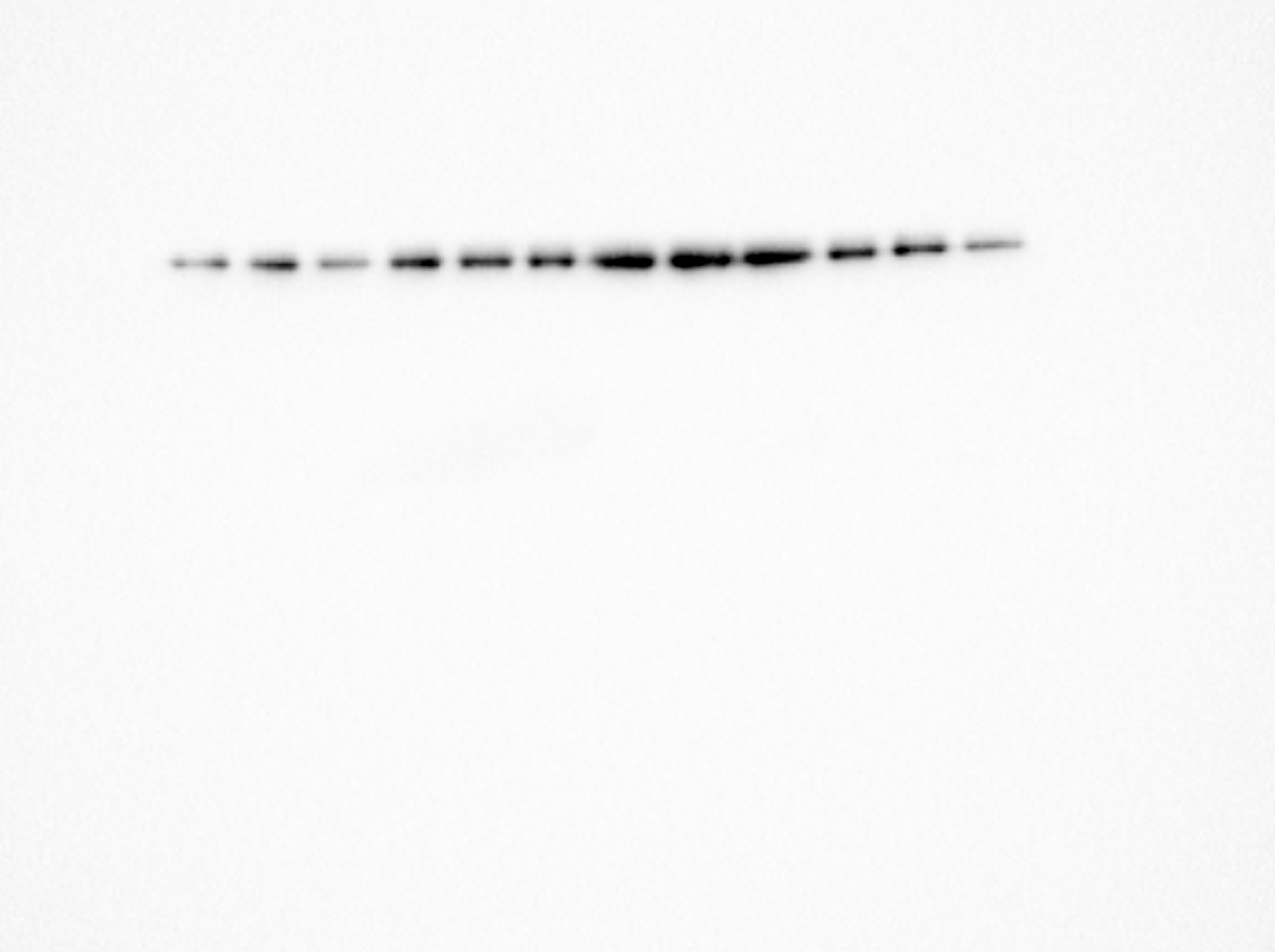

Supplement: Supplementary file 1 [file DataSheet_1.zip › Raw data-2021-12-18/Raw data-Western Blot-2021-12-18/CONíóATRAíóTGEVíóT+ATRA/RLRs/RIG-1.jpg]

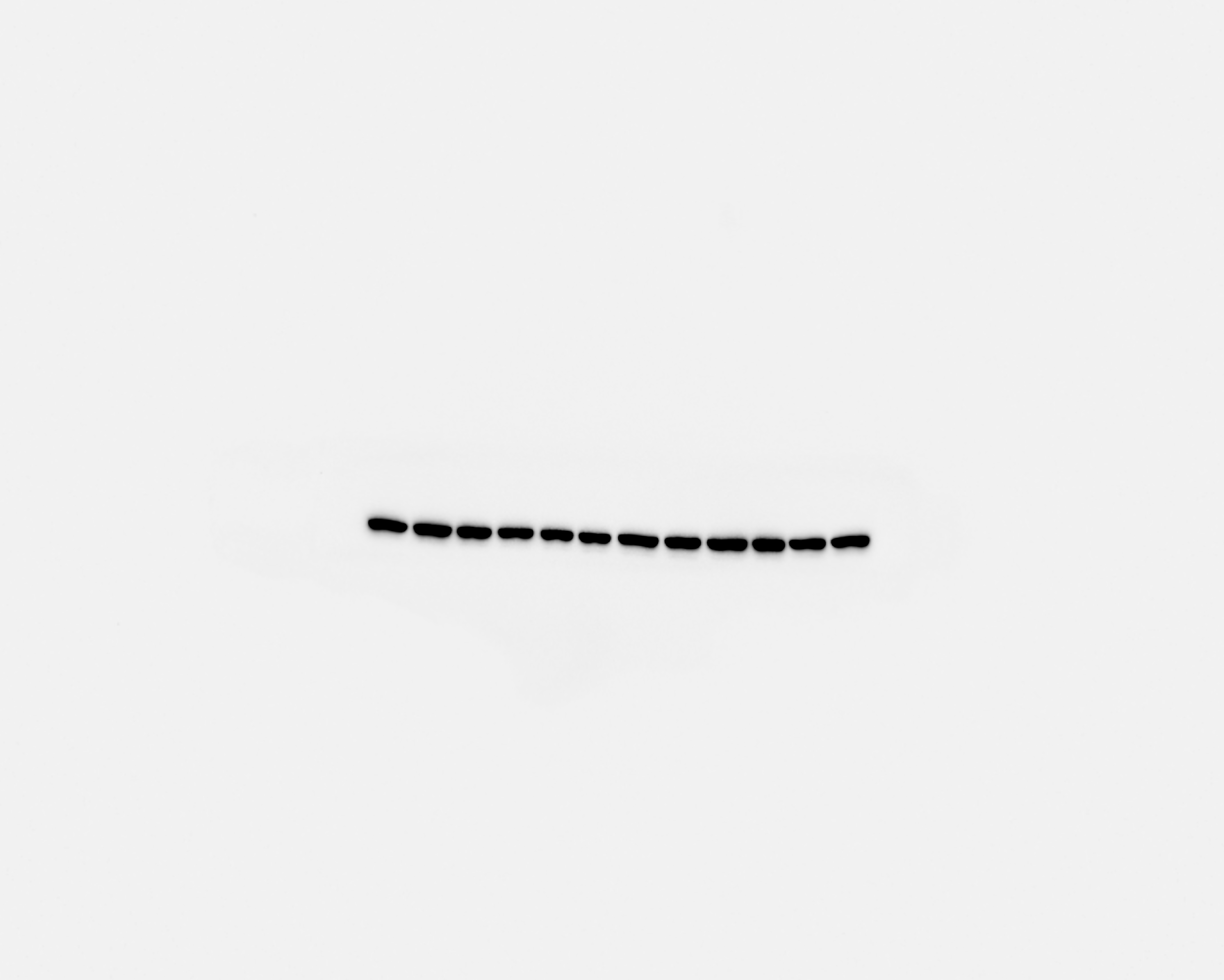

Supplement: Supplementary file 1 [file DataSheet_1.zip › Raw data-2021-12-18/Raw data-Western Blot-2021-12-18/CONíóATRAíóTGEVíóT+ATRA/RLRs/a┬-actin.jpg]

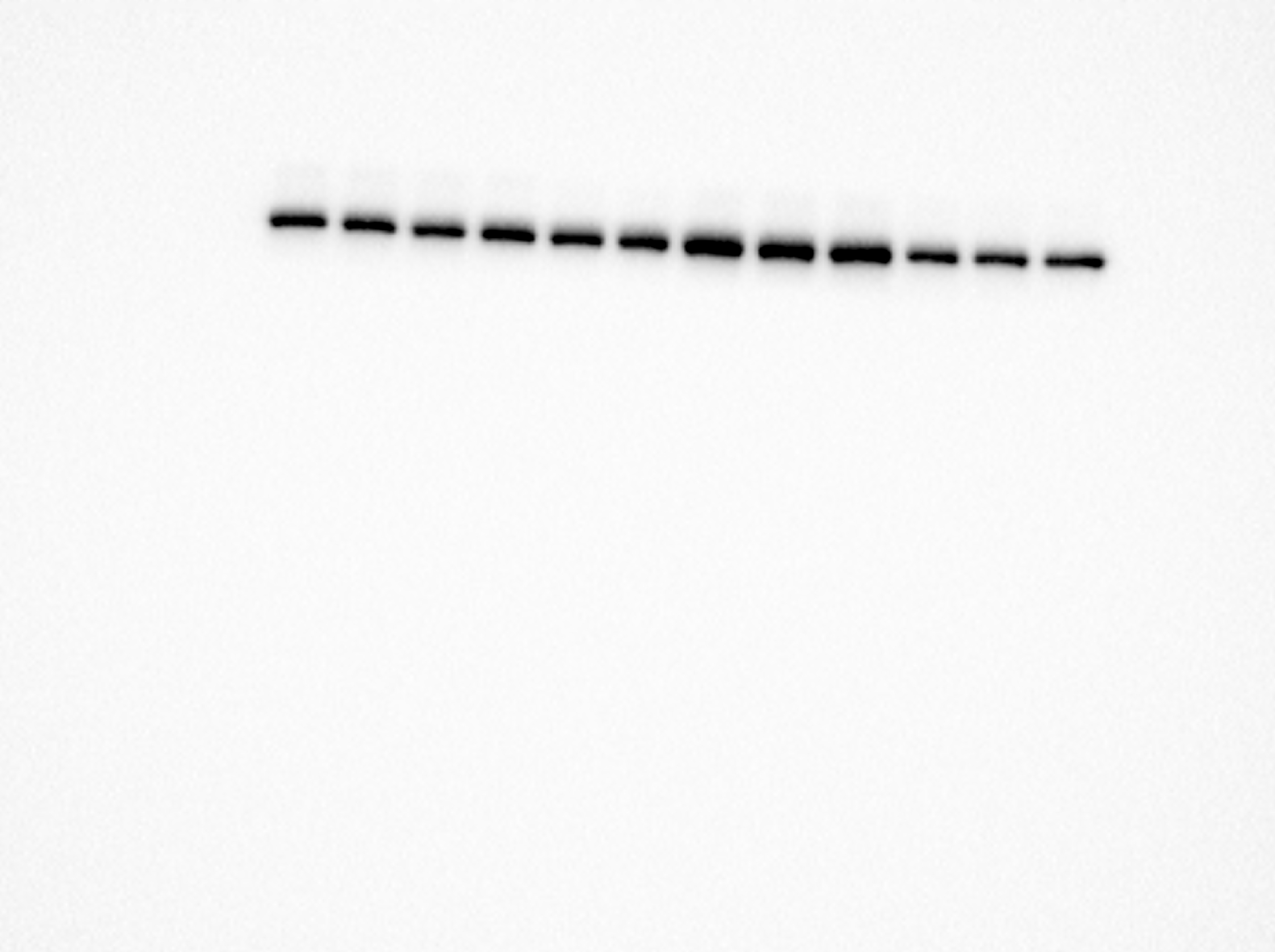

Supplement: Supplementary file 1 [file DataSheet_1.zip › Raw data-2021-12-18/Raw data-Western Blot-2021-12-18/CONíóATRAíóTGEVíóT+ATRA/TLRs/TLR3.jpg]

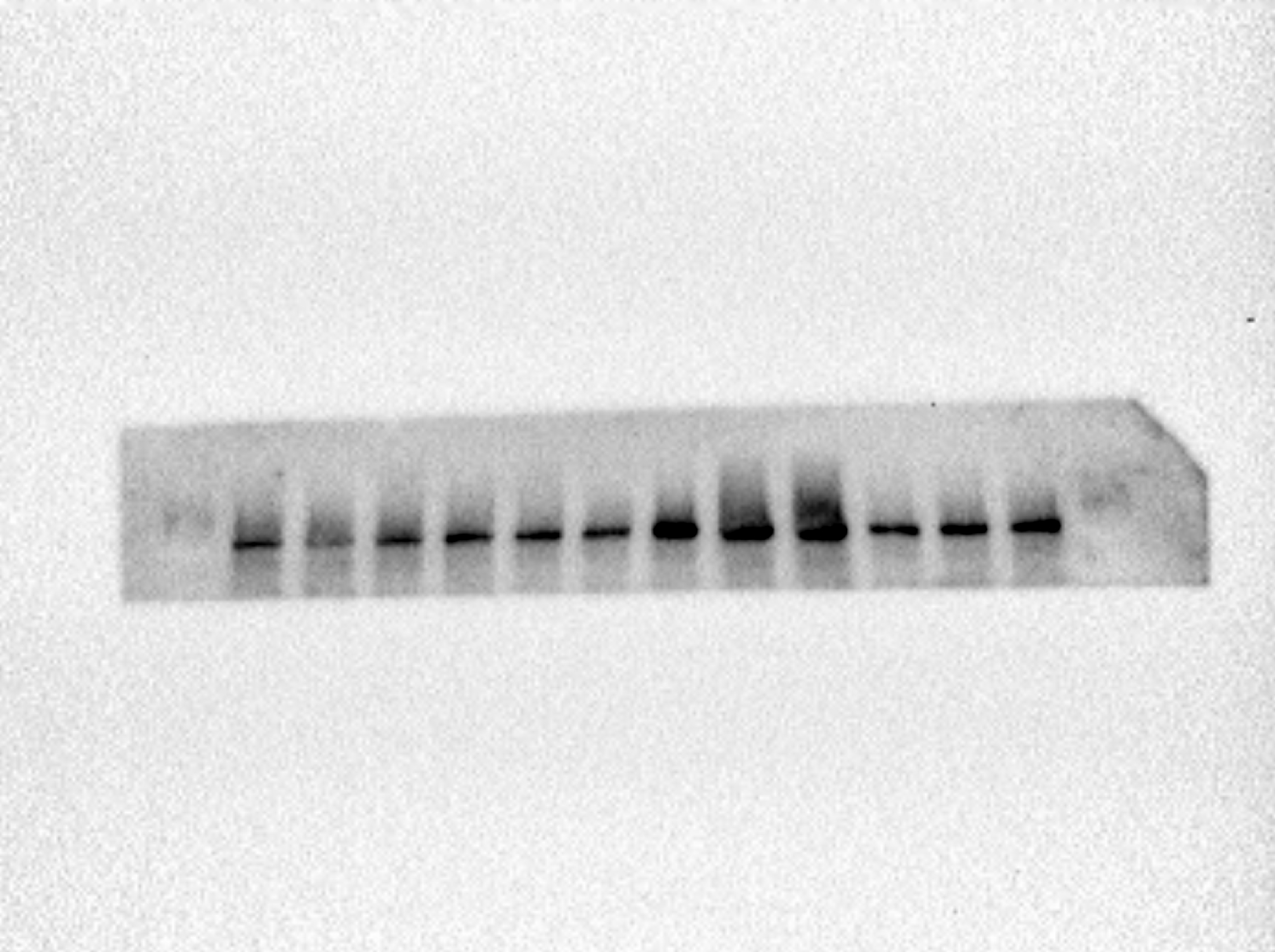

Supplement: Supplementary file 1 [file DataSheet_1.zip › Raw data-2021-12-18/Raw data-Western Blot-2021-12-18/CONíóATRAíóTGEVíóT+ATRA/TLRs/TLR7.jpg]

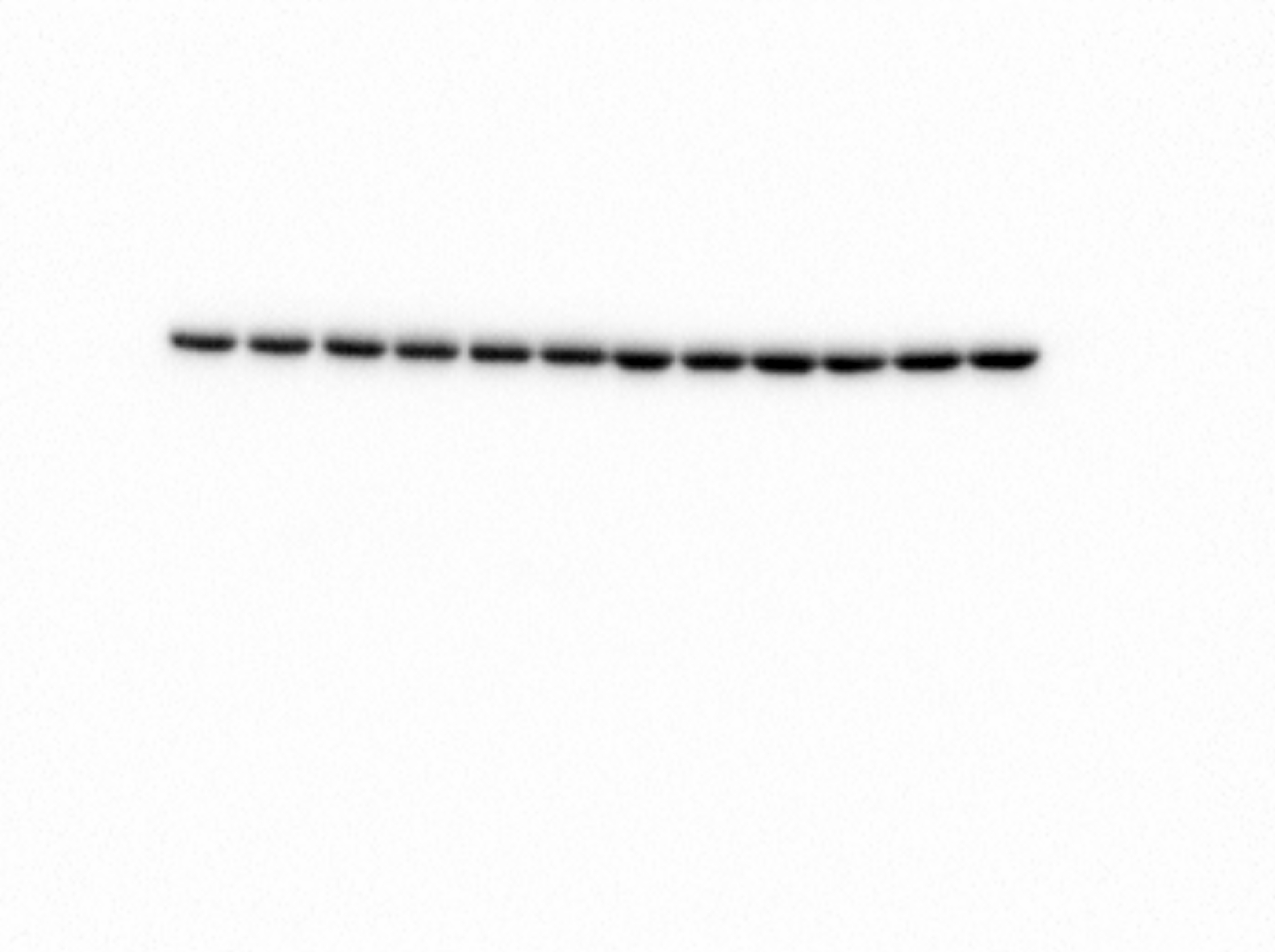

Supplement: Supplementary file 1 [file DataSheet_1.zip › Raw data-2021-12-18/Raw data-Western Blot-2021-12-18/CONíóATRAíóTGEVíóT+ATRA/TLRs/a┬-actin.jpg]

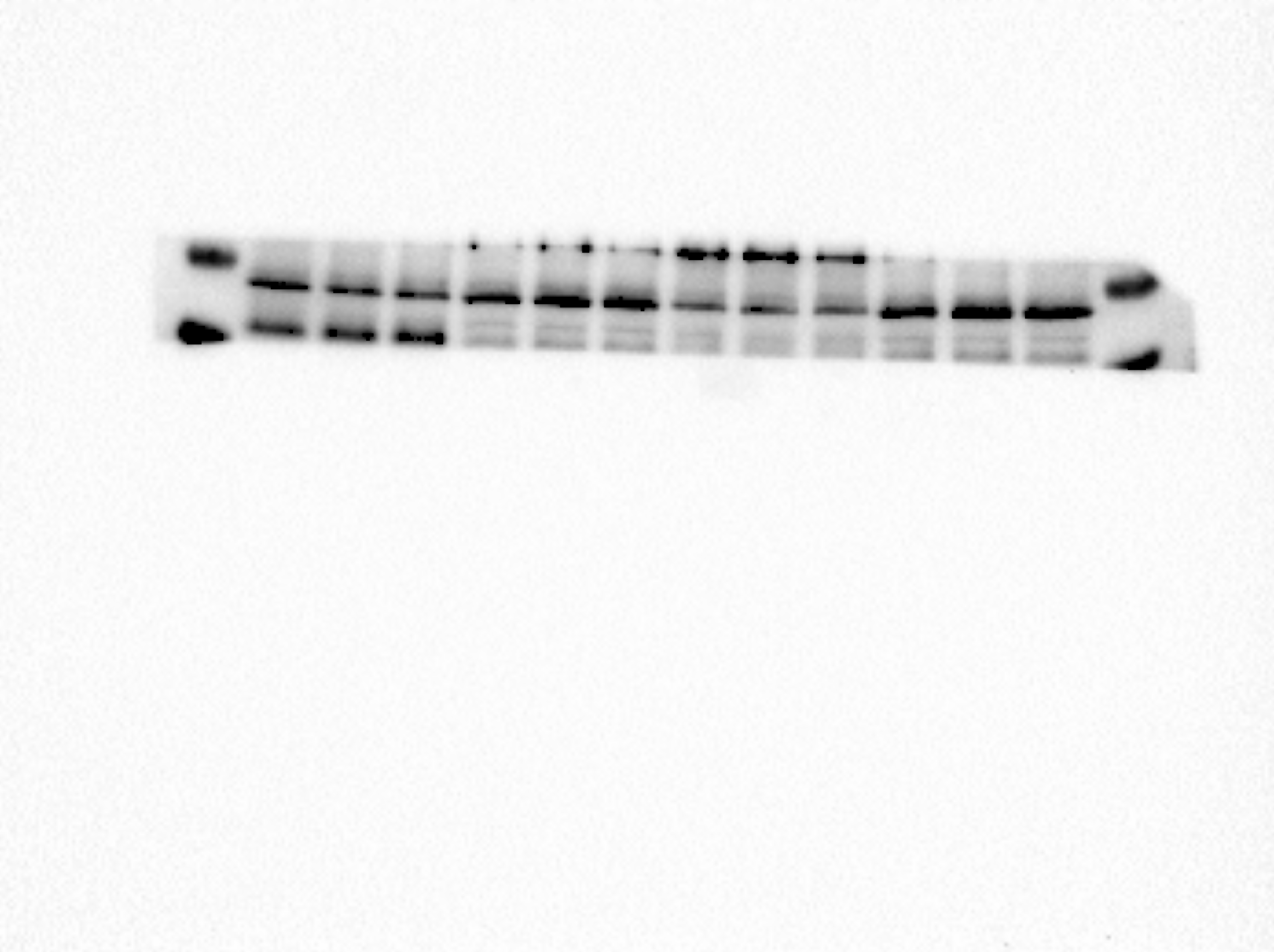

Supplement: Supplementary file 1 [file DataSheet_1.zip › Raw data-2021-12-18/Raw data-Western Blot-2021-12-18/CONíóATRAíóTGEVíóT+ATRA/Tight junction proteins/Occludin.jpg]

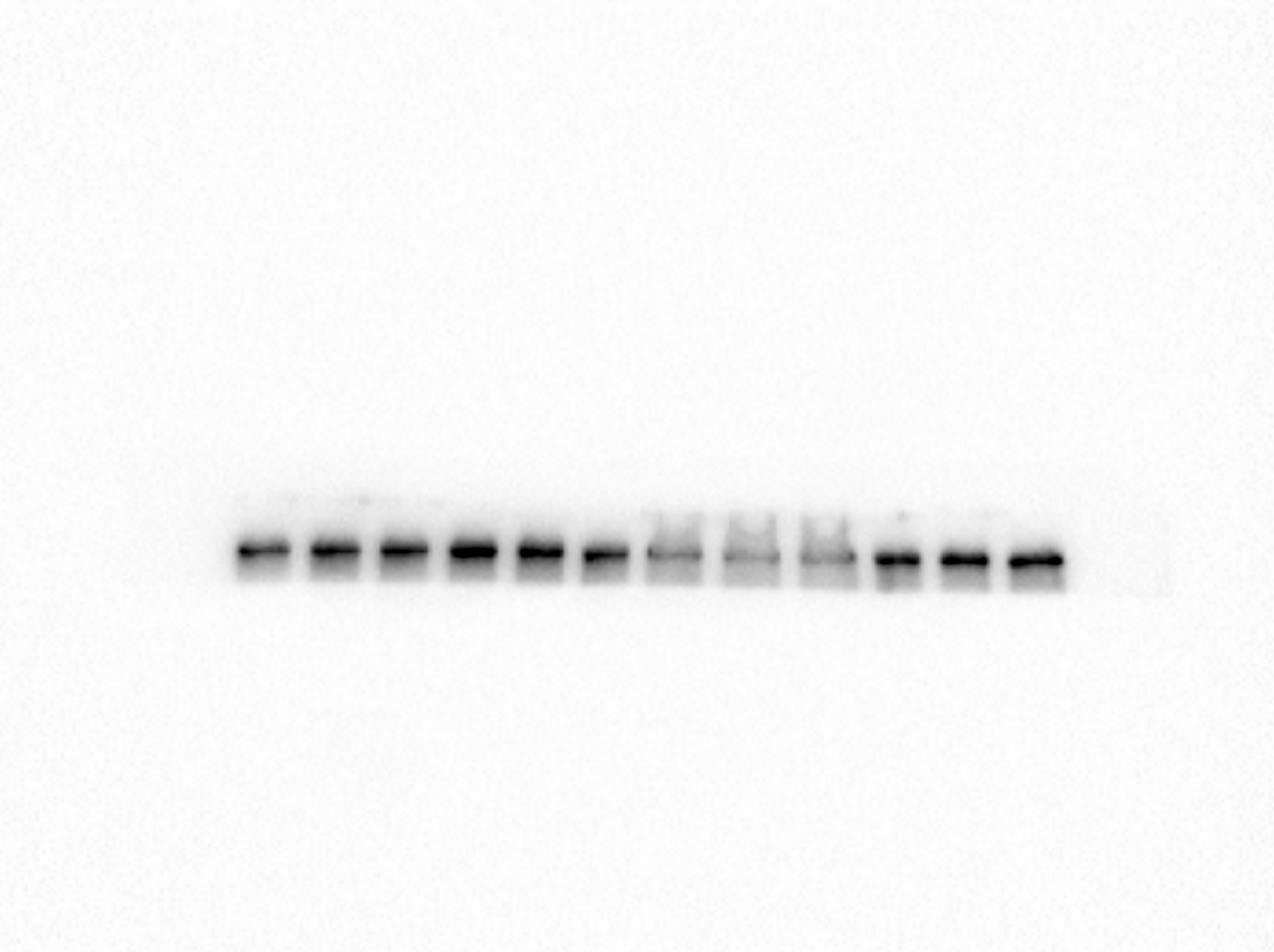

Supplement: Supplementary file 1 [file DataSheet_1.zip › Raw data-2021-12-18/Raw data-Western Blot-2021-12-18/CONíóATRAíóTGEVíóT+ATRA/Tight junction proteins/ZO-1.jpg]

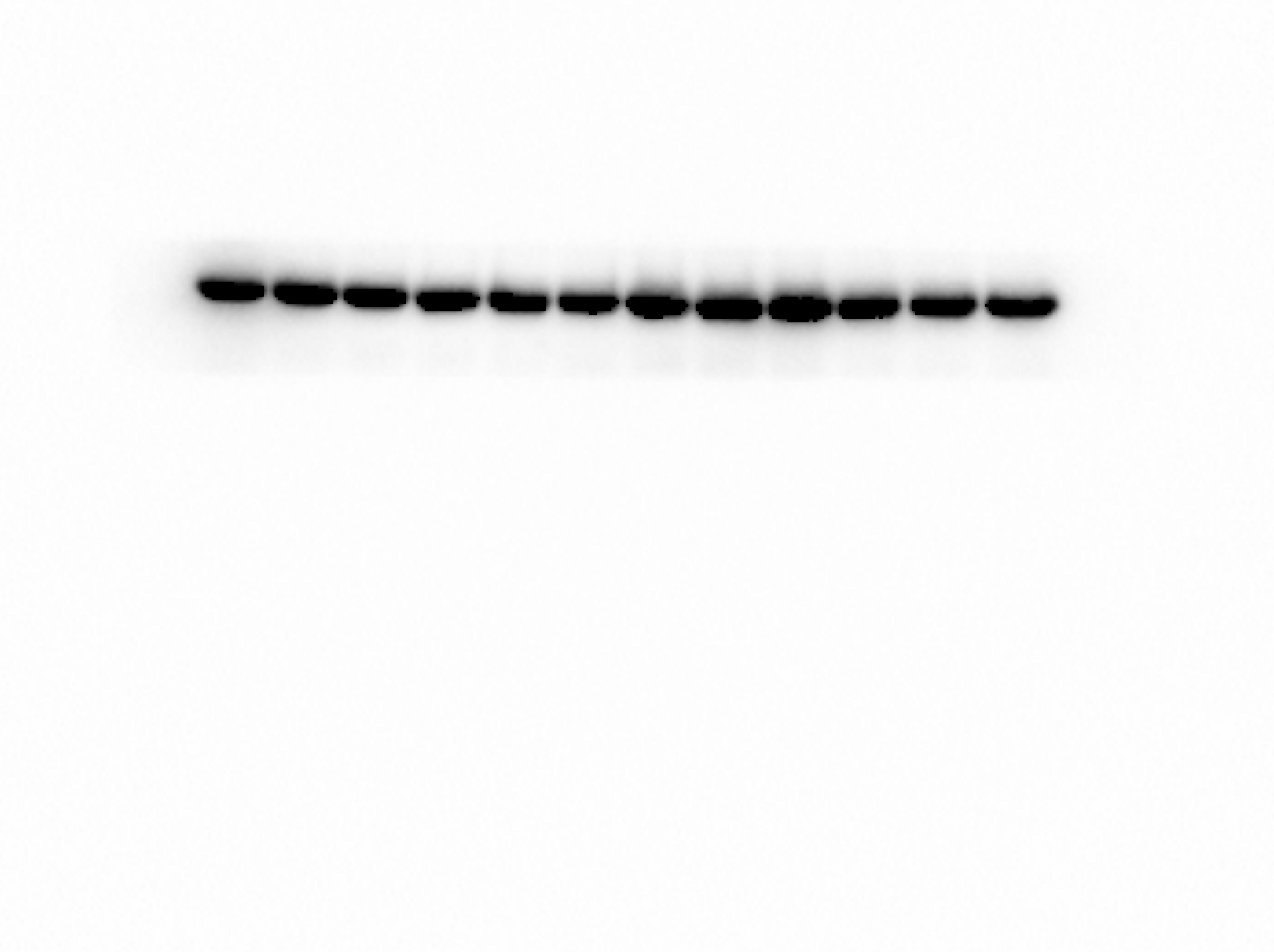

Supplement: Supplementary file 1 [file DataSheet_1.zip › Raw data-2021-12-18/Raw data-Western Blot-2021-12-18/CONíóATRAíóTGEVíóT+ATRA/Tight junction proteins/a┬-actin.jpg]

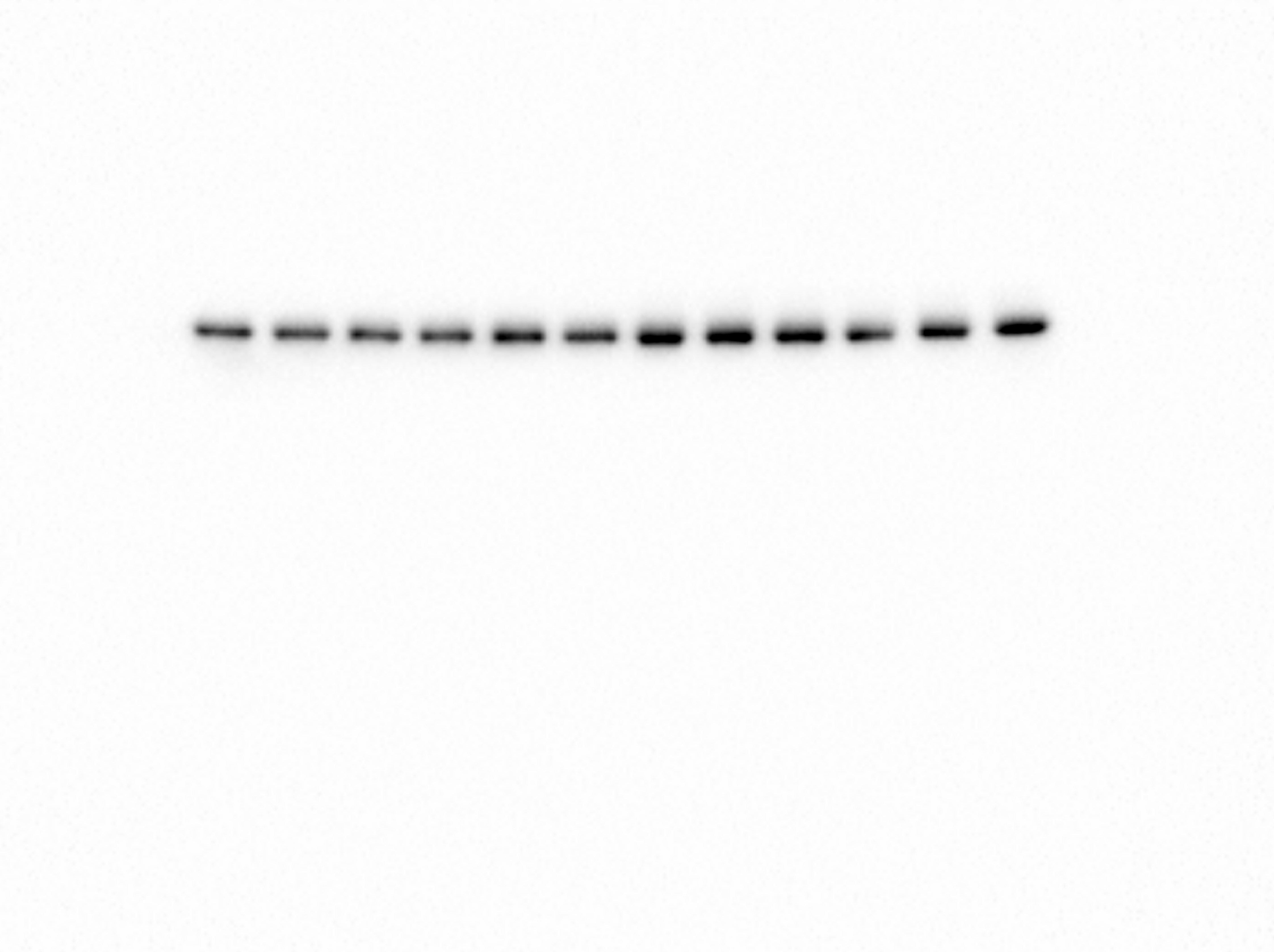

Supplement: Supplementary file 1 [file DataSheet_1.zip › Raw data-2021-12-18/Raw data-Western Blot-2021-12-18/CONíóTGEVíóT+ATRAíóT+BAY/NF-a╩B p65.jpg]

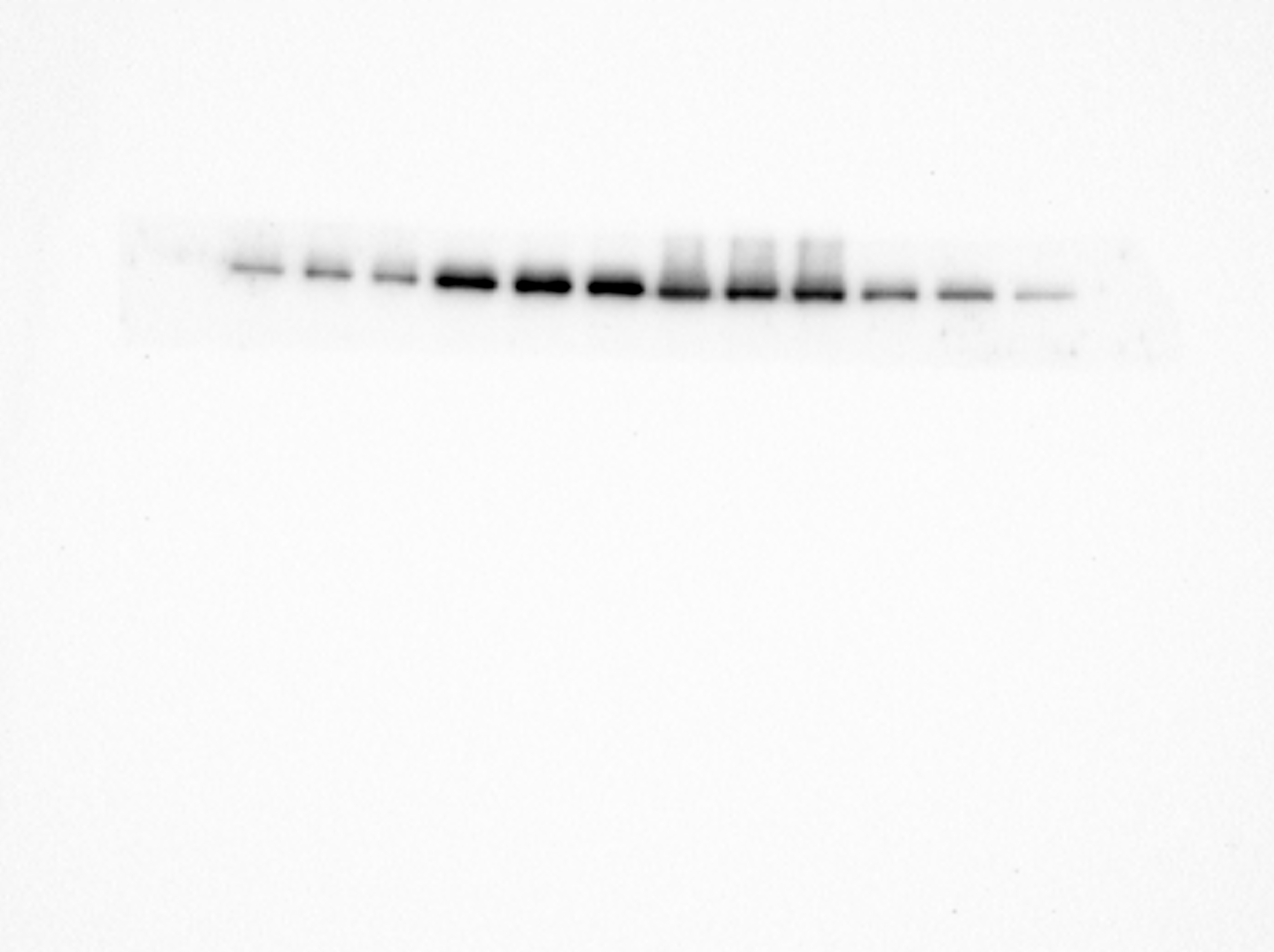

Supplement: Supplementary file 1 [file DataSheet_1.zip › Raw data-2021-12-18/Raw data-Western Blot-2021-12-18/CONíóTGEVíóT+ATRAíóT+BAY/p-NF-a╩B p65.jpg]

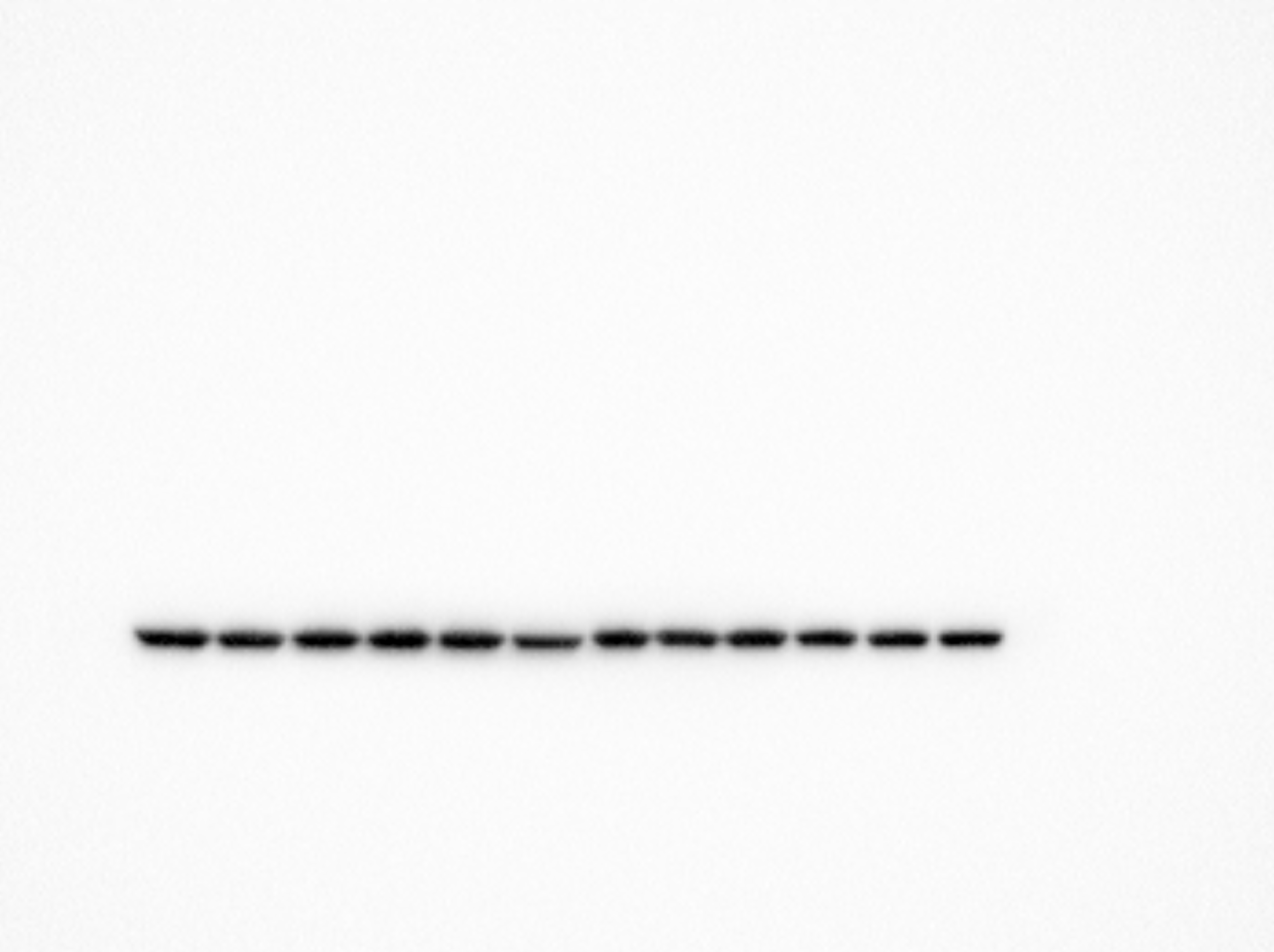

Supplement: Supplementary file 1 [file DataSheet_1.zip › Raw data-2021-12-18/Raw data-Western Blot-2021-12-18/CONíóTGEVíóT+ATRAíóT+BAY/a┬-actin.jpg]

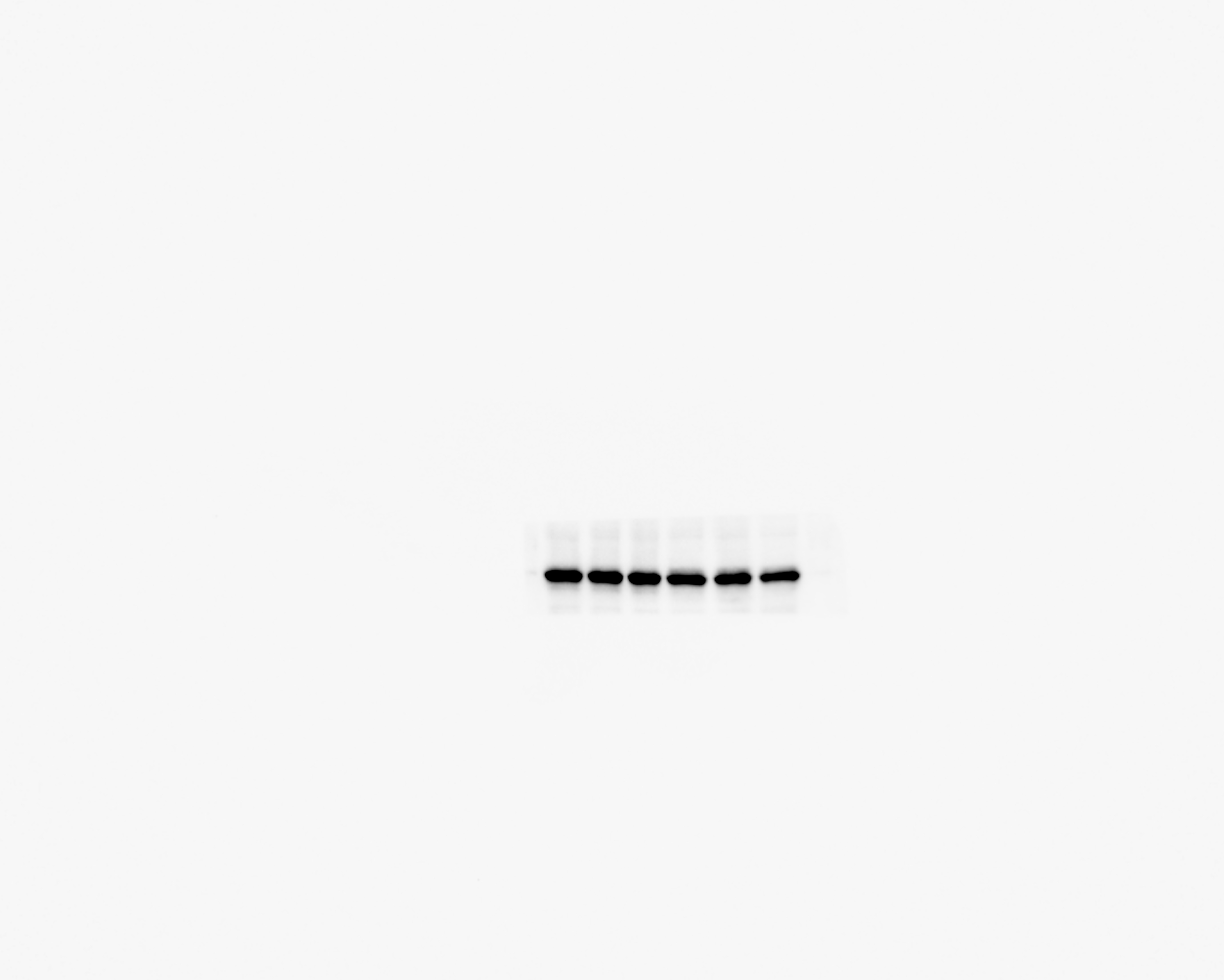

Supplement: Supplementary file 1 [file DataSheet_1.zip › Raw data-2021-12-18/Raw data-Western Blot-2021-12-18/CONíóUV-TGEV/NF-KB p65.jpg]

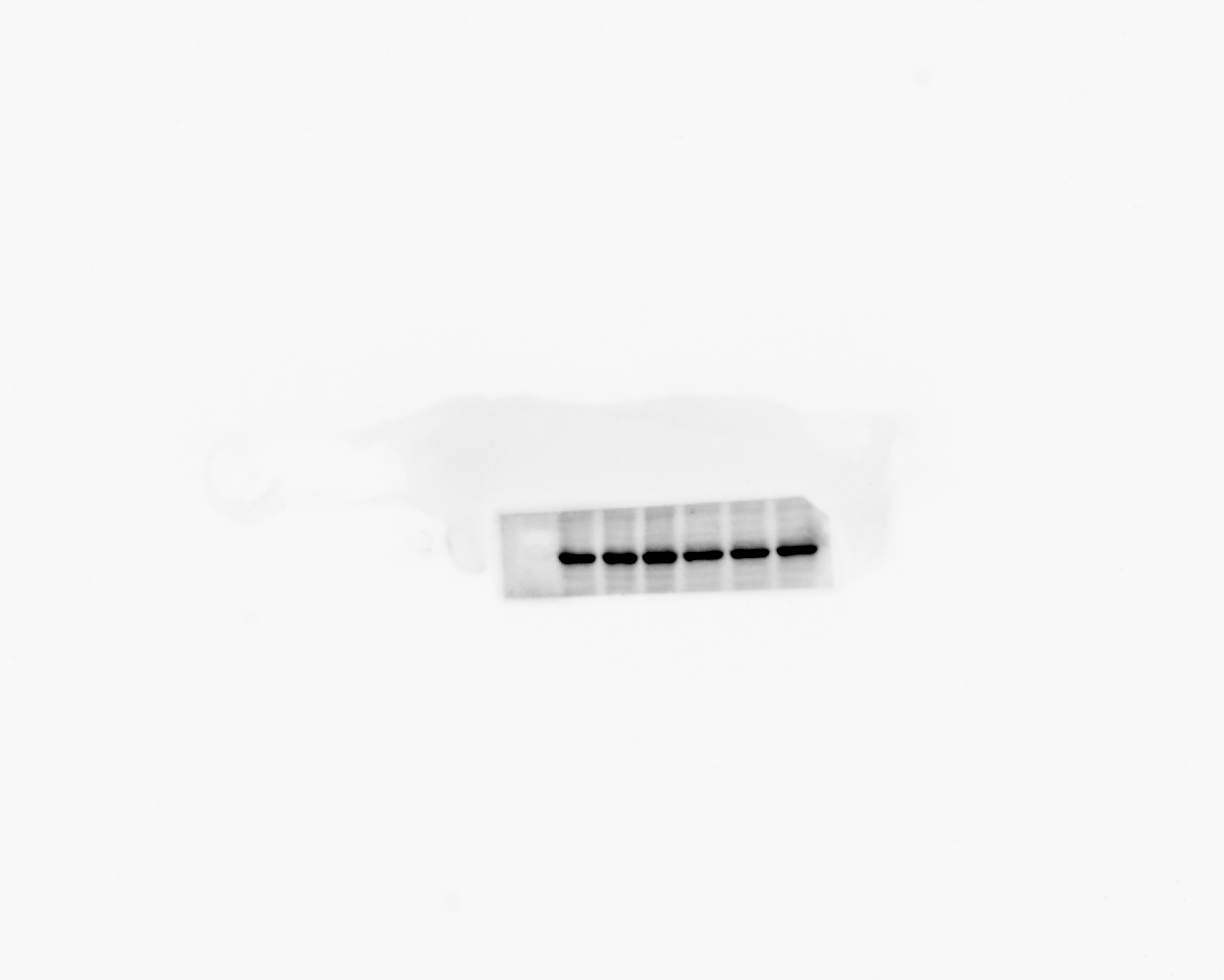

Supplement: Supplementary file 1 [file DataSheet_1.zip › Raw data-2021-12-18/Raw data-Western Blot-2021-12-18/CONíóUV-TGEV/p-NF-KB p65.jpg]

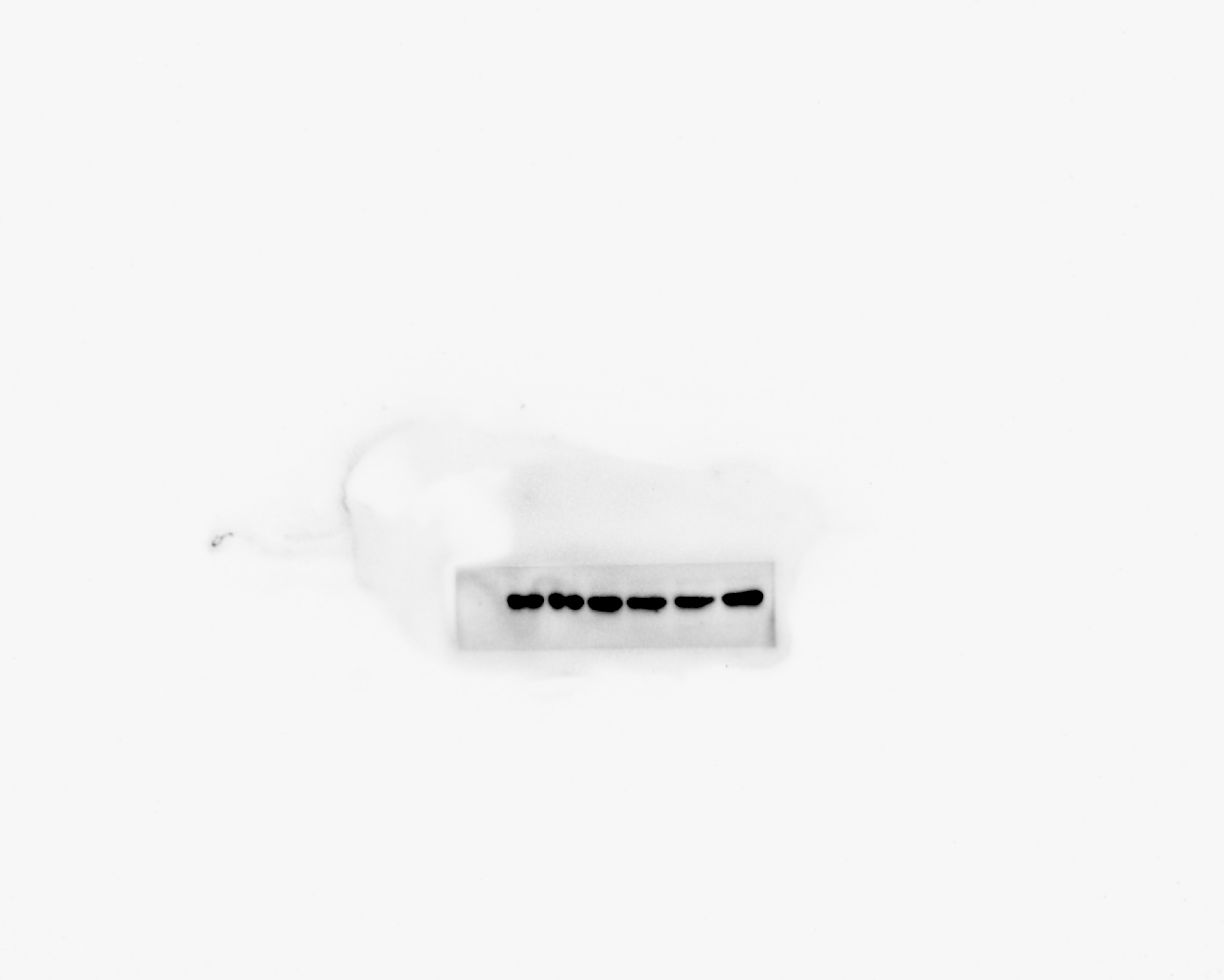

Supplement: Supplementary file 1 [file DataSheet_1.zip › Raw data-2021-12-18/Raw data-Western Blot-2021-12-18/CONíóUV-TGEV/a┬-actin.jpg]

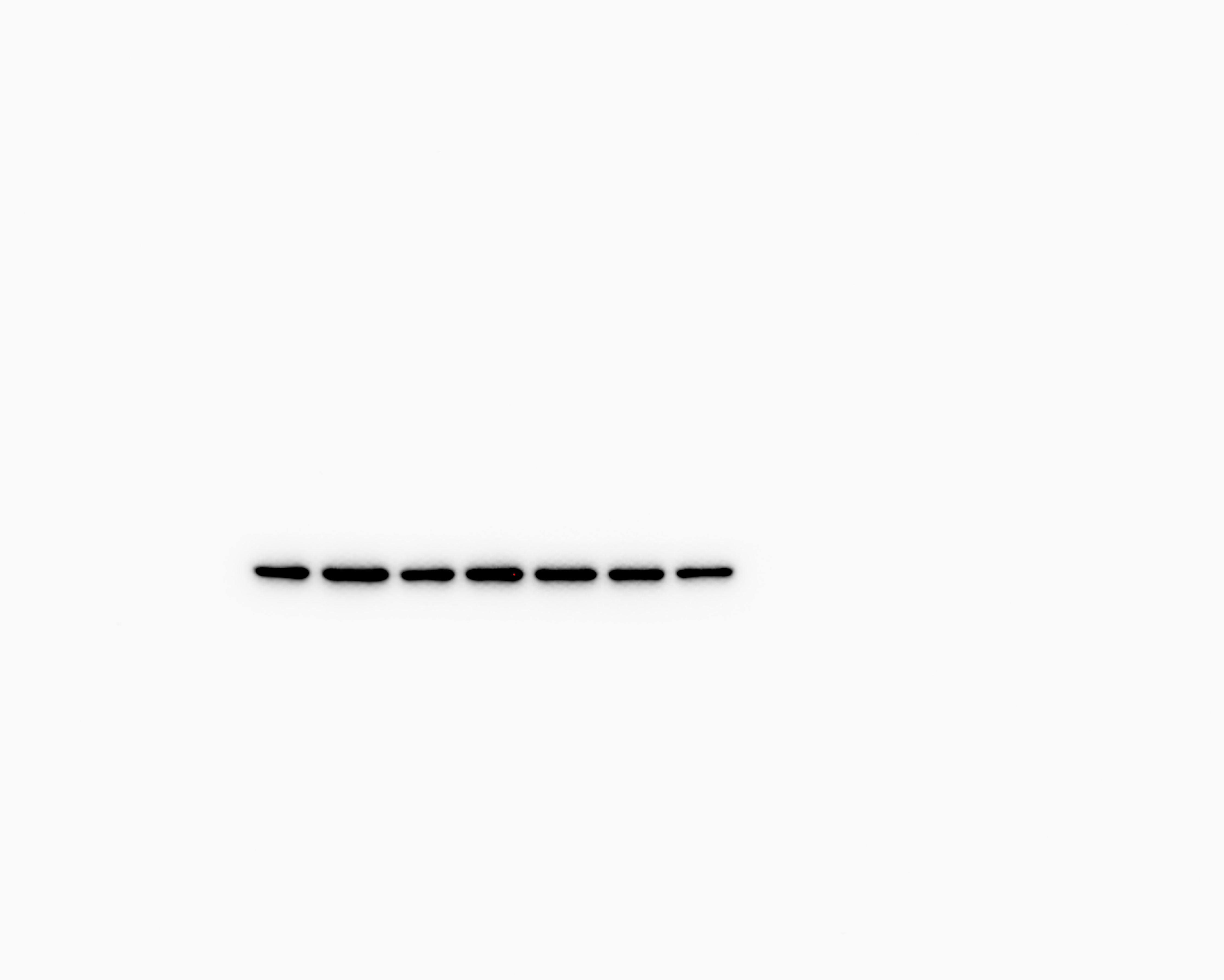

Supplement: Supplementary file 1 [file DataSheet_1.zip › Raw data-2021-12-18/Raw data-Western Blot-2021-12-18/siNCíóT+siNCíóT+ATRAíóT+siTLR3íóT+siTLR7íóT+siRIG-1íóT+siMDA5/NF-a╩B p65.jpg]

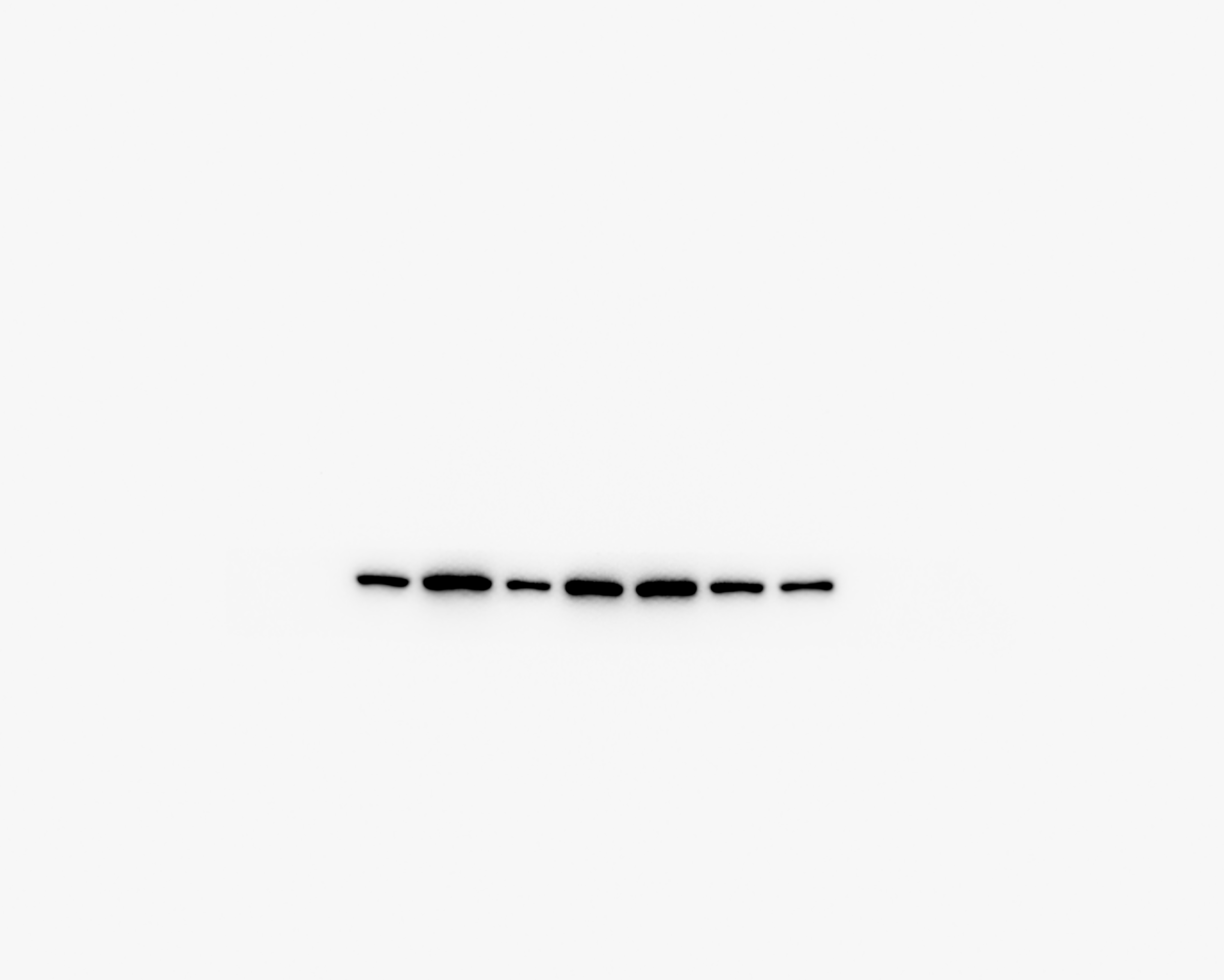

Supplement: Supplementary file 1 [file DataSheet_1.zip › Raw data-2021-12-18/Raw data-Western Blot-2021-12-18/siNCíóT+siNCíóT+ATRAíóT+siTLR3íóT+siTLR7íóT+siRIG-1íóT+siMDA5/p-NF-a╩B p65.jpg]

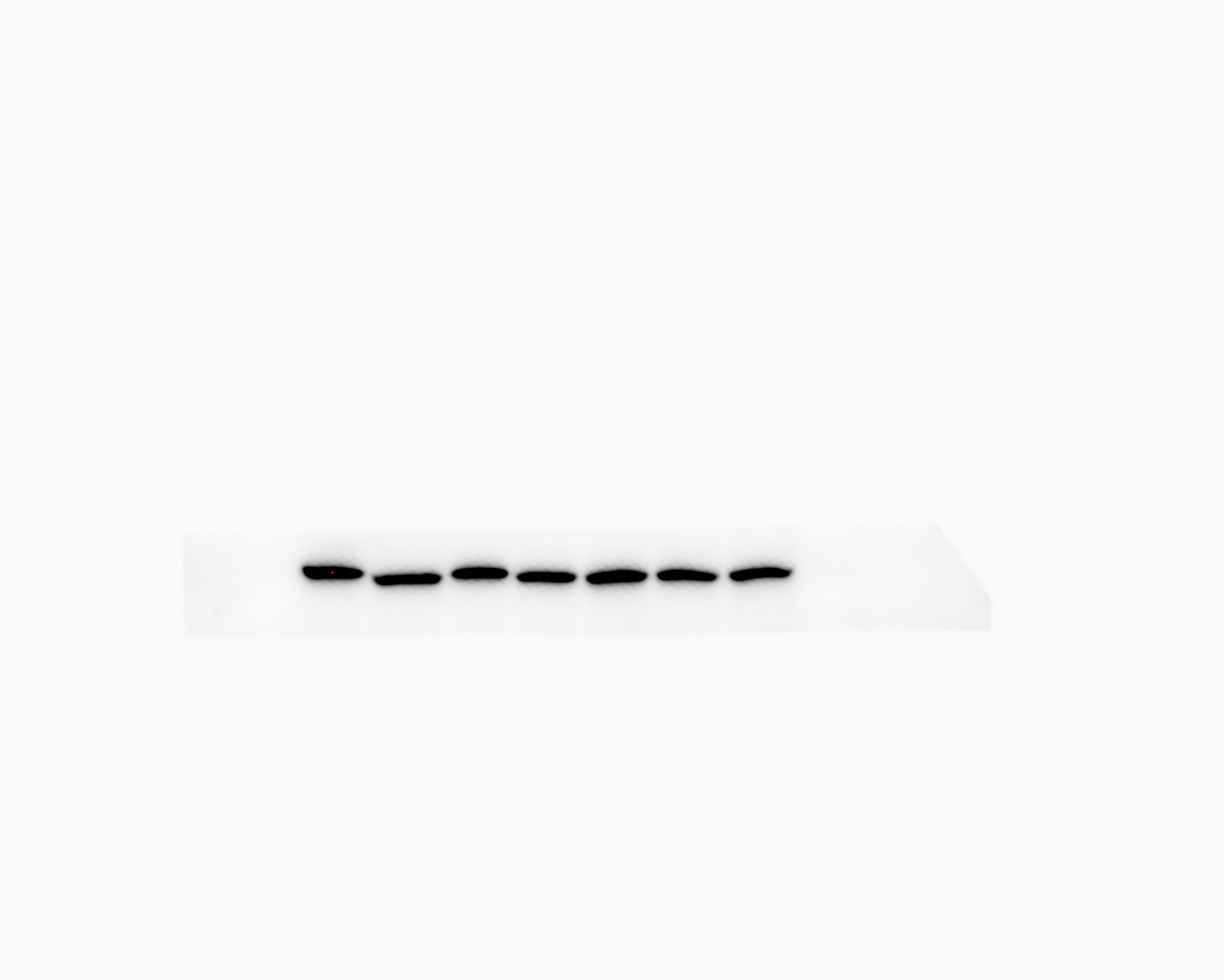

Supplement: Supplementary file 1 [file DataSheet_1.zip › Raw data-2021-12-18/Raw data-Western Blot-2021-12-18/siNCíóT+siNCíóT+ATRAíóT+siTLR3íóT+siTLR7íóT+siRIG-1íóT+siMDA5/a┬-actin.jpg]
